# PATTERNA: transcriptome-wide search for functional RNA elements via structural data signatures

**DOI:** 10.1186/s13059-018-1399-z

**Published:** 2018-03-01

**Authors:** Mirko Ledda, Sharon Aviran

**Affiliations:** 1 Department of Biomedical Engineering and Genome Center, UC Davis, 1 Shields Ave, Davis, 95616 USA; 2 Integrative Genetics and Genomics Graduate Group, UC Davis, 1 Shields Ave, Davis, 95616 USA

**Keywords:** RNA structure, Structure probing, Machine learning, Pattern recognition, Functional elements, Transcriptome-wide profiling, SHAPE, PARS, RNA structure–function, GMM-HMM

## Abstract

Establishing a link between RNA structure and function remains a great challenge in RNA biology. The emergence of high-throughput structure profiling experiments is revolutionizing our ability to decipher structure, yet principled approaches for extracting information on structural elements directly from these data sets are lacking. We present patteRNA, an unsupervised pattern recognition algorithm that rapidly mines RNA structure motifs from profiling data. We demonstrate that patteRNA detects motifs with an accuracy comparable to commonly used thermodynamic models and highlight its utility in automating data-directed structure modeling from large data sets. patteRNA is versatile and compatible with diverse profiling techniques and experimental conditions.

**Electronic supplementary material:**

The online version of this article (10.1186/s13059-018-1399-z) contains supplementary material, which is available to authorized users.

## Background

RNA plays a central role in various aspects of a cell’s machinery, from gene expression and regulation to protein synthesis [1, 2]. While an RNA sequence is informative, it is widely accepted that its functionality is directly attributable to the formation of specific and intricate secondary and tertiary structures, highlighting the importance of accurate structure models at high resolution [3]. High-resolution RNA structures can be obtained experimentally using X-ray crystallography [4] or nuclear magnetic resonance [5], or computationally using comparative sequence analysis [6, 7]. However, these methods have shortcomings, most notably in terms of cost or manual labor, rendering them low throughput. To circumvent these limitations, models of folding energetics, such as the nearest-neighbor thermodynamic model (NNTM) [8, 9], have been used to predict secondary structure computationally from sequence information. Despite their popularity, predicted structures generally suffer from poor accuracy [10], especially when applied in an in vivo context or to long RNAs. Additional insights into RNA structure have been gleaned from structure profiling (SP) experiments, which recently emerged as an affordable and high-throughput approach to structure analysis. SP methods provide snapshots of structural states at nucleotide resolution in vivo or in vitro and shed light on the role of structures in governing biological functions [2].

SP experiments can be performed in many ways. However, they all aim at interrogating structural characteristics, at nucleotide resolution, for all RNAs in a sample while relying on common principles [11, 12]. To this end, SP methods utilize chemical reagents or enzymes that are sensitive to the local stereochemistry in the vicinity of a nucleotide and result in the formation of chemical adducts or cleavage events on the RNA backbone [13–21]. Consequently, these reagents induce either terminations or mutations during reverse transcription, enabling detection of modifications by primer extension analyses. The resulting cDNA products undergo sequencing to quantify termination or mutation events, which are then converted into a reaction rate for each nucleotide. The advent of next-generation sequencing has scaled this paradigm to the transcriptome-wide level and has also resulted in a plethora of available techniques [11]. Methods differ in choice of probing reagent and strategies for sample and library preparation, modification detection, and data analysis. Consequently, they generate data with disparate statistical properties. To date, one can profile the structure of an entire transcriptome, called the structurome, both in vitro and in vivo, which opens up new opportunities for data-driven structure–function studies. For instance, these methods have revealed significant insights into the key role of structure in regulating the transcriptomes of bacteria, yeasts, *Arabidopsis*, mice, and humans [11].

These new capabilities are revolutionizing our ability to decipher structures, link them to biological functions, and harness them to engineer novel functional RNAs, with new applications constantly emerging [2, 22–28]. However, to date, principled and universal approaches for interpreting and mining information from SP data sets are severely lacking, with experimentalists resorting to methods tailored ad hoc to each experiment and biological question [26, 29]. For instance, no general method has been developed for harnessing SP data to search for user-specified functional elements at the transcriptome scale. At present, such elements are found by leveraging homologies between a sought-after target motif and transcriptomic regions. In these approaches, motifs take the form of sequence or predicted structures [3, 30–39], or combinations thereof [40–42]. While powerful, they rely on structure modeling assumptions that fail to capture the full complexity of a cell, including biomolecular interactions and changing cellular conditions, which commonly affect the stability of structures. Importantly, these aspects are captured by SP data. Notable examples of regulatory elements for which consensus structure models exist and which are also impacted by cellular conditions include RNA regions that respond to ligands (aptamers and riboswitches) [43–45] or temperature (thermosensors) [46, 47], G-quadruplexes [48, 49], and other non-coding RNAs [50]. Very recently, it also became evident that epitranscriptomic modifications, known to affect structure stability, are far more dynamic and prevalent than previously thought [51, 52]. Moreover, SP data are valuable when gauging structural kinetics, which are notoriously difficult to predict in silico [53–57]. For example, SP was recently used to elucidate cotranscriptional folding pathways in vitro [23] and in vivo at a transcriptome-wide scale [28]. To infer the kinetics, the experimentalists resorted to manual inspection and qualitative comparisons of reactivities at select nucleotides, which they combined with prior knowledge to model structural rearrangements. Henceforth, the discovery of new functional structures depends upon the development of new approaches that transcend traditional prediction algorithms and interpret SP data within the context of a functional domain as opposed to individual nucleotides [26].

Bridging the gap between an RNA’s structure and its biological function critically requires statistically sound methods and novel computational frameworks for analyzing and interpreting SP data [58–62]. In that context, we describe PATTERNA, a fast pattern recognition algorithm that mines user-specified RNA structure motifs in SP data sets. Our approach is based on a Gaussian mixture model-hidden Markov model (GMM-HMM) statistical learning framework, inspired by automated speech recognition, which captures key properties of SP signals in any given data set. PATTERNA learns parameters for the model directly from the data without relying on knowledge of the underlying structures, whereas existing data-directed methods must be trained from experiment-specific structure profiles of RNAs with known reference structures [29, 63, 64]. Once trained, the model is used exclusively for structural inference, thereby presenting an additional advance by transcending the current thermodynamics-based structure prediction paradigm [26]. Its NNTM-free approach also confers PATTERNA with the flexibility to mine complex structural elements, such as pseudoknots (PKs) and self-contained tertiary interactions. Overall, PATTERNA’s design principles render it a reference-free and NNTM-free method, which is versatile and compatible with virtually all SP techniques conducted at any scale and probing conditions, from a handful of RNAs probed in vitro to transcriptome-wide in vivo data sets. We demonstrate that it accurately detects structural motifs in real and diverse data sets at multiple scales and that it facilitates and automates structure inference in a dynamic biological system. Our findings highlight that PATTERNA aids in gaining structural insights into complex systems, which we believe will greatly accelerate the discovery of novel functional RNAs.

## Results

### Overview of statistical model and inference

**Fig. 1:**
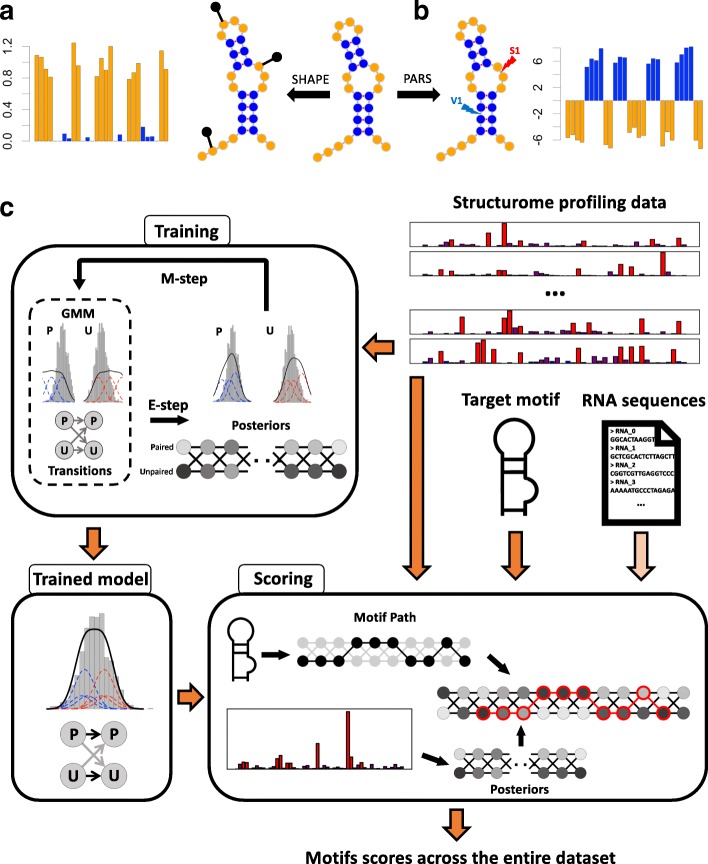
Overview of structure profiling data and PATTERNA. **a, b** Schematic representation of an RNA secondary structure with paired and unpaired nucleotides highlighted in blue and orange, respectively. Structure diagrams were obtained with Forna [100]. **a** SHAPE experiments entail structure-dependent formation of chemical adducts, indicated by black pins on the RNA, which are subsequently detected by sequencing and used to produce a reactivity for each nucleotide. High/low reactivities correspond to unpaired/paired nucleotides. **b** PARS experiments use two nucleases: RNAse S1 cleaves single-stranded RNA while RNAse V1 cleaves double-stranded RNA. Cleavage sites are detected by sequencing and summarized into a single score, where negative/positive scores indicate unpaired/paired nucleotides. **c** Cartoon overview of PATTERNA. PATTERNA is trained on input structure profiles using an iterative expectation-maximization algorithm that learns the statistical properties of nucleotide pairing states and the data distributions associated with each pairing state. The illustrated GMM model uses three Gaussian components per pairing state. Once trained, PATTERNA can be applied to the same transcripts used for training or to new transcripts. The scoring phase uses the structure profiling data and the trained model to infer the posterior probabilities of each pairing state, which are then used to score the state sequence that represents the motif. Motifs are scored across all starting nucleotides and input transcripts. Optionally, sequence constraints can be applied to restrict the search to regions that permit the formation of the motif’s base pairs. GMM Gaussian mixture model, P paired, U unpaired

At the core of PATTERNA is a GMM-HMM that learns the statistical properties of RNA secondary structures from SP data alone. Key to our model is a representation of secondary structure as a sequence of nucleotide pairing states, where each nucleotide assumes one of two states: paired or unpaired. This is a simplification of the standard notion of structure, which is defined by a list of base-pairing partners and unpaired nucleotides. Here, we do not require any knowledge of pairing partners because SP data does not reveal them (Fig. 1a, b). The objective of our method is to detect a user-specified structural motif across a data set.

PATTERNA runs in two distinct phases: training and scoring. During training, we fit the GMM-HMM to the SP data using the Baum–Welch algorithm, an iterative expectation-maximization (EM) algorithm that maximizes the likelihood of the data given the model (Fig. 1c). The GMM part captures SP data properties, specifically, the data distributions associated with each pairing state, which describe the probability of observing a value given the underlying pairing state. The HMM part models unknown (hidden) pairing states and the probability of transitioning from one to another. This intuitively results in learning general RNA structure characteristics. For instance, very long stretches of unpaired nucleotides are unlikely to occur in real structures. Once PATTERNA is trained, the scoring phase can be accomplished on either the same data set used for training or a new input data set. The first step in scoring is to use our trained model to estimate pairing state probabilities, for each nucleotide, from the input data. These probabilities and the most likely state sequence given the trained model (Viterbi path) can be requested as an output. Since SP collects data at the nucleotide level, whereas motifs span stretches of nucleotides, we must bridge the resolution of measurements and that of sought-after patterns. To accomplish this, a motif is encoded as a binary sequence of pairing states (the motif path) and the trained GMM-HMM is used to estimate the probability of the motif, given the data, across input transcripts. RNA sequences can be additionally provided, in which case PATTERNA outputs only regions whose sequence permits the formation of Watson–Crick and Wobble base pairs that are present in the motif. Complete details are available in “Methods” section and in Additional file 1.

### Automated reference-free learning of structure from profiling data

**Fig. 2:**
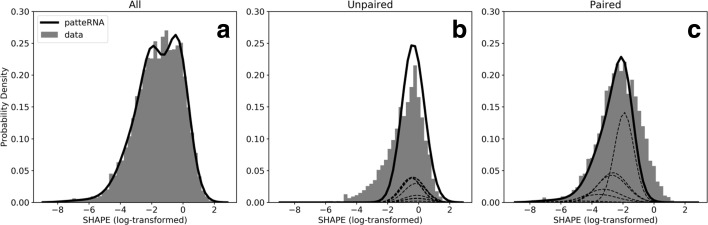
Model of SHAPE data from the Weeks set. **a** Log-transformed SHAPE reactivities (grey bars) were fitted by PATTERNA using a Gaussian mixture model (black line) summed across ten Gaussian components per state. Reactivities were subsequently broken down into each pairing state using reference structures to assess the accuracy of PATTERNA’s state-dependent models at unpaired (**b**) and paired (**c**) nucleotides. Dashed lines correspond to individual Gaussian components

To test if our framework accurately models real data without reference structures, we used a curated data set of 21 RNAs with known structures and with SHAPE profiles from the Weeks lab [59, 65, 66]. This data set, hereafter called the Weeks set, consists of highly structured non-coding RNAs (Additional file 2: Table S1) and therefore, does not represent a typical transcriptome composition. Nevertheless, it provides a ground truth of pairing states against which we can benchmark our model-based predictions. We tested our framework with both raw and log-transformed reactivities using ten Gaussian components per state. We log-transformed the data because we previously showed that log-transformed data are approximated well by a normal distribution, and that this eases and standardizes the statistical treatment of such data [64]. Figure 2a and Additional file 2: Figure S1A–C show that PATTERNA fits both reactivity distributions with high fidelity. To investigate if PATTERNA can model state-dependent distributions, we used the pairing information provided by reference structures to partition the data into two distinct distributions for unpaired and paired nucleotides [29, 63]. Our results indicate that PATTERNA is able to approximate these distributions, even in the absence of reference structures (Fig. 2b,c). To determine the stability of our estimates with respect to random initialization and the number of Gaussian components (see “Methods” section), we repeated the training 100 times on log-transformed data and using models ranging from 1 to 25 Gaussian components. Our results indicate strong agreement between fitted models, suggesting that while the log-likelihood of PATTERNA may be non-convex, therefore, not guaranteeing a universal unique solution, in practice, we achieve stable estimates with well-behaved data (Additional file 2: Figure S2).

Having established that PATTERNA effectively learns the statistical properties of SP signals directly from the data, we next assessed its ability to call nucleotide pairing states correctly. We considered both the posterior probabilities of pairing states as well as the most likely secondary structure given our model, which we generated using the Viterbi algorithm (see “Methods” section). In our benchmark, we included minimum free energy (MFE) state sequences predicted by RNAprob [64] as well as state sequences predicted from SHAPE data alone using a cutoff-based classifier that uses an optimized threshold to classify SHAPE reactivities into paired and unpaired states. The threshold is optimized with respect to the reference structures. To accommodate posteriors and the optimized cutoff classifier, we chose the *ℓ*1-norm distance between predicted and reference structures as an accuracy metric. Our results show that we obtain similar accuracies between raw and log-transformed SHAPE data for both the optimized cutoff classifier and PATTERNA (Table 1), suggesting that our framework produces consistent outputs even when the shapes of the data distributions differ significantly. Furthermore, we observed no major differences in accuracy between PATTERNA’s Viterbi path, its posterior path, and the optimized cutoff classifier. This is encouraging because both PATTERNA and the optimized cutoff classifiers do not make any thermodynamic modeling assumptions but rather rely solely on SP data to draw inferences. However, the latter is also informed by the true structural states in the reference structures. As such, it signifies the maximal information that can be extracted from SHAPE data alone, thus the comparable accuracies suggest that our model generates near-optimal posterior estimates.

**Table 1:** Accuracy of predicted secondary structures using the Weeks set

	Raw		Log transformed		Fragmented	
	*ℓ*_1_-norm	Accuracy [%]	*ℓ*_1_-norm	Accuracy [%]	*ℓ*_1_-norm	Accuracy [%]
Optimized cutoff	2843	73.0	2810	73.3	–	–
patteRNA - Viterbi	2916	72.3	2939	72.1	–	–
patteRNA - posteriors	2962.3	71.9	2981.9	71.7	–	–
MFE	2406	77.2	–	–	2964	71.9
MFE+SP	1890	82.1	–	–	2588	75.4

*MFE* minimum free energy, *SP* structure profiling

Overall, the best performance is obtained using NNTM-based predictions, with SHAPE-directed predictions providing the best results. This is expected, as this approach is informed by both folding thermodynamics modeling and SP data. However, when we generated MFE structures using 100 nt (nucleotides) fragments in place of full-length sequences to mimic strategies used for transcriptome-wide searches using NNTM-based methods, the performance using MFE structures dropped significantly. Under these conditions, MFE predictions from sequence alone perform comparably to the optimized cutoff classifier and PATTERNA while data-driven MFE predictions maintain a smaller advantage. Overall, our results demonstrate that structural information can be gleaned from SHAPE data directly, thereby obviating the need for reference structures.

### Detecting canonical motifs

**Fig. 3:**
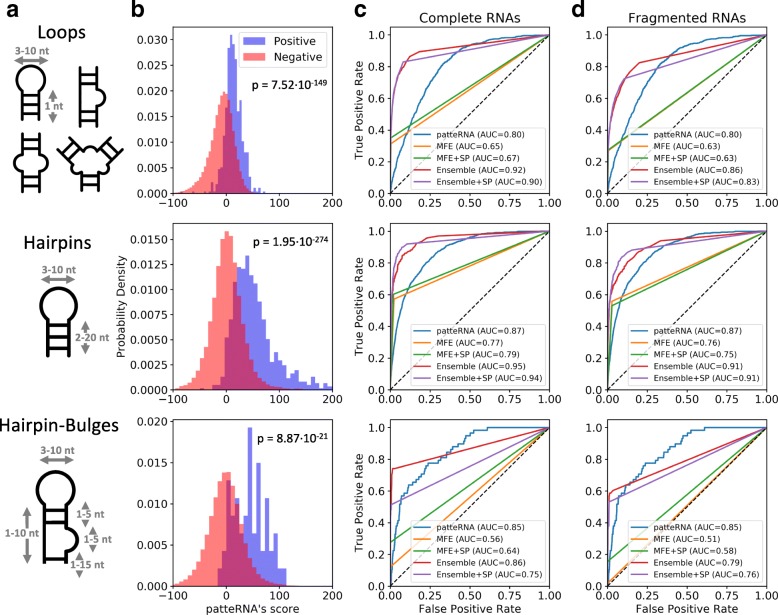
PATTERNA accurately detects canonical motifs in SHAPE data. The performances of five methods with the Weeks SHAPE data set are compared: PATTERNA, MFE structure prediction using NNTM (MFE), data-directed MFE structure prediction using NNTM (MFE+SP), Boltzmann ensemble sampling (Ensemble), and data-directed Boltzmann ensemble sampling (Ensemble+SP). **a** Schematic secondary structures of three scored motifs (loops, haipins, and hairpin-bulges). Allowed variations in loop and stem lengths are indicated by grey double arrows. **b**PATTERNA’s score histograms at regions where the tested motif was present (positive) or absent (negative) in reference structures. The *p* value corresponds to a one-sided Mann–Whitney *U* test between the two distributions. **c, d** ROC curves of performance of motif detection for each method when applied to complete (**c**) and fragmented (**d**) RNAs. The area under the curve (AUC) is reported in the legend. The dashed lines correspond to the performance expected from a random classifier. AUC area under the curve, MFE minimum free energy, NNTM nearest-neighbor thermodynamic model, ROC receiver operating characteristic, SP structure profiling

The primary goal of our work is to detect a user-defined structural motif rapidly in SP data. To evaluate PATTERNA’s detection accuracy, we searched for various RNA motifs in the Weeks set. We started by identifying loops ranging from 3 to 10 nt and padded on each side by at least one paired nucleotide (Fig. 3a). The true presence or absence of loops was determined from reference structures. To compare our results, we performed a similar search on MFE structures predicted by RNAprob as well as 1000 structures statistically sampled from a Boltzmann ensemble by GTfold [67] (see “Methods” section for details). For both methods, we compared sequence-only as well as SHAPE-directed predictions. The Weeks set contains 619 loops that match our size constraints and PATTERNA scored regions containing a loop significantly better than regions that did not contain one (Fig. 3b, one-sided Mann–Whitney *Up* value =7.52×10^−149^). Receiver operating characteristic (ROC) analysis further showed that the best accuracy is obtained by ensemble sampling, with or without SHAPE constraints [Fig. 3c, ROC area under the curve (AUC) = 0.90–0.92]. Interestingly, our results also reveal that PATTERNA (AUC = 0.80) sensibly outperforms MFE predictions (AUC = 0.65–0.67) (Fig. 3c). We next searched for two additional motifs: a single hairpin (stem: 2–20 nt, loop: 3–10 nt) and a similar hairpin followed by a small bulge (1–5 nt) (Fig. 3a). Since these motifs do not involve long-range interactions and can be folded locally, we also used the RNA sequences to exclude regions where motifs could not form. The set contains 792 matching hairpins, and again, PATTERNA scored regions containing the target motif significantly better than other regions, with Mann–Whitney *Up* value =1.95×10^−274^ and =8.87×10^−21^ for hairpins and hairpin-bulge motifs, respectively (Fig. 3b). The best performance is attained by ensemble sampling, followed by PATTERNA, while MFE predictions fall short (Fig. 3c). Similarly, for the hairpin-bulge composite motif, present 58 times in the set, PATTERNA shows substantially higher detection accuracy compared to MFE prediction, highlighting a weakness of the NNTM whose parameters might not be universally accurate [68] (Fig. 3c).

Overall, these results are not surprising as an ensemble-based approach considers many competing alternative structures while an MFE approach encapsulates structural dynamics into a single structure [69]. Nevertheless, our results also indicate that PATTERNA performs surprisingly well, given that no sequence information is used, and can even outperform thermodynamics modeling on occasions. These results would initially suggest that an ensemble-based method should be used to detect structural motifs. However, the computational burden associated with sampling the ensemble, which is cubic in the length of the RNA [i.e., $\mathcal {O}\left (n^{3}\right)$ for length *n*], renders transcriptome-scale analysis impractical due to the multitude of long transcripts that must be considered.

For practical examples, we used runtime benchmarks with simulated data sets and included PATTERNA, GTfold, ViennaRNA [70, 71], and RNAstructure [72] (see Additional file 2: “Runtime benchmarks,” Figure S3, and Tables S4–S7). We used simulations to examine runtime dependence both on RNA length and data set size. Results show that GTfold, the fastest ensemble-sampling method we tested, would take over a year to process a single transcriptome-wide data set (Additional file 2: Table S7). To equip users with the option to pre-evaluate time requirements readily for their data sets, we further provide a formula to estimate the time requirements for large data sets using these popular NNTM prediction algorithms (see Additional file 2: “Runtime benchmarks” and Table S5). In addition, long RNAs (>1000 nt) generally require deeper sampling of the ensemble, compared to short RNAs, to ensure that all biologically relevant structures are represented in the sampled pool and that sample frequencies reliably approximate their theoretical counterparts. Therefore, this approach is typically reserved for small-scale studies of small RNAs [69], or alternatively, for small targeted regions within transcripts (e.g., 100 nt long), as these are easily folded computationally. MFE prediction suffers a similar drawback given its complexity is also $\mathcal {O}\left (n^{3}\right)$ (Additional file 2: Figure S3) and, while faster compared to ensemble sampling, it would still require about a month for a typical transcriptome-wide data set (Additional file 2: Table S7). Therefore, for both methods, at transcriptome scale, one must either restrict the analysis to a manageable subset of the data or perform motif searches in the absence of the entire sequence context of an RNA, for example within 100-nt windows to minimize the burden associated with computational complexity. To emulate this method, we fragmented each RNA in the Weeks set into 100-nt segments and repeated our entire analysis. As expected, PATTERNA’s performance was closer to ensemble-based predictions on the Weeks set (Fig. 3d). Importantly, it should be noted that the impact of folding locally, i.e., without considering the entire sequence context, on prediction performance is currently poorly characterized and may also be case-specific.

In summary, we demonstrated the feasibility of detecting a user-specified structural motif in SHAPE data with no additional information. In other words, we can relax constraints based on transcript sequences, effectively alleviating the requirement for complex and resource-demanding NNTM-based secondary structure predictions, while having a relatively small impact on the performance of motif detection. Furthermore, because we do not use a thermodynamic model, the computational complexity of motif detection is reduced by two orders of magnitude compared to alternative NNTM-based methods, such that it is linear in RNA length, $\mathcal {O}(n)$, even without sequence constraints (Additional file 2: Figure S3). At such complexity, large transcriptome-wide data sets can be processed within a few days at worst, compared to months or years with NNTM-based methods (Additional file 2: Table S7). Also note that PATTERNA is trained only once for each data set considered, following which it can be used to score as many motifs as required. Although EM algorithms sometimes suffer from slow convergence, PATTERNA does not need to be trained on entire data sets. A small subset of transcripts (<1000) with a high data density and quality will have sufficiently robust data and structure characteristics that generalize to the entire data set. An additional shortcoming of thermodynamics modeling is its inability to consider inter- or intra-molecular interactions, which stabilize or destabilize particular secondary structures or motifs. Such interactions are common in vivo, yet they are largely absent in the in vitro conditions in which the Weeks set was obtained. This highlights another advantage of making predictions from SP data alone, especially in complex cellular environments. We, thus, expect PATTERNA to generate even more accurate predictions when applied to in vivo data. Moreover, note that the Weeks data set contains RNAs for which NNTM-based predictions are remarkably improved when directed by SHAPE data [59, 64, 65]. However, this does not generalize to all RNAs, as performance gains for data-driven NNTM predictions can vary significantly [63]. It, thus, remains unclear how data-driven NNTM performs across large sets of diverse RNAs.

### Motif detection in a bi-stable regulatory system

**Fig. 4:**
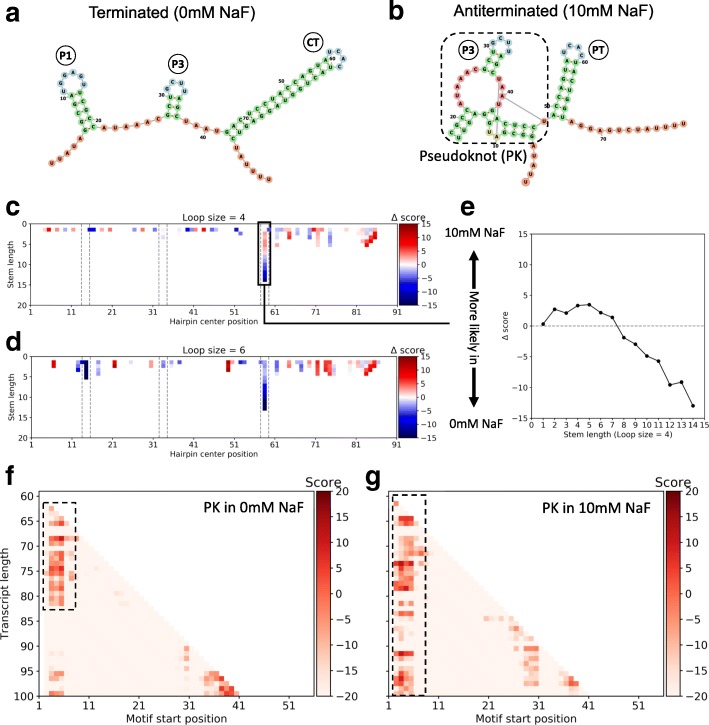
Motif detection in a bi-stable fluoride riboswitch. **a** The accepted structure of the terminated fold, associated with the transcription off state, features three hairpins: P1, P3, and CT (complete terminator). **b** The accepted structure of the anti-terminated fold, associated with the transcription on state, features a pseudoknot domain (dashed box) and a partial terminator (PT) hairpin. **c, d** Differential scores between fluoride conditions. Hairpins of loop size 4 and 6 and variable stem size were scored across all possible starting nucleotides of the full-length transcript (100 nt). The *x*-axis indicates the position of the motif’s start. The *y*-axis corresponds to hairpins with stems of variable lengths. As sequence constraints were applied, only regions that permit base pairings in the stem were scored. Reds indicate that the motif is more likely in 10 mM NaF, and blues that it is more likely in 0 mM NaF. Dashed lines highlight the location of the P1, P3, and PT/CT hairpins. **e** Differential scores at the terminator hairpin site as a function of increasing stem size. **f, g** Pseudoknot scored at each possible starting nucleotide and across all intermediate transcripts without (**f**) and with (**g**) fluoride. Reds indicate higher scores. Dashed boxes highlight regions where a pseudoknot is likely present. PK pseudoknot

To investigate further our ability to detect structural motifs from SP data, we used in vitro cotranscriptional SHAPE-seq data collected from the *Bacillus cereus* fluoride riboswitch—an RNA domain that changes conformation upon binding of a small molecule—in the absence and presence of 10 mM NaF [23]. In this method, protein roadblocks are embedded into DNA templates and terminate transcription, henceforth producing intermediate transcripts that are subsequently profiled by SHAPE-seq [73]. This series of increasingly longer profiles can be used to infer folding trajectories of elongating transcripts. The conformational changes triggered upon fluoride binding involve extensive structural rearrangements of hairpin motifs (Fig. 4a, b) [74, 75]. In the absence of NaF, three consecutive hairpins form, but upon binding of a fluoride anion, the first helix (P1) unwinds in favor of the formation of a PK, whereas the third helix (P3) is greatly shortened. Such a dynamic environment is ideal for testing PATTERNA because the RNA sequence remains fixed across conditions and therefore, structural differences can be gleaned only from the data.

As before, we used ten Gaussian components per state when training our model (Additional file 2: Figure S1D). We started by searching for hairpins of variable sizes, with loop size ranging from 4 to 6 nt and stem size ranging from 1 to 20 nt, while enforcing sequence constraints (results without sequence constraints are available in Additional file 2: Figure S4). We then conducted a differential analysis between conditions by subtracting the score of each hairpin in 0 mM NaF from its score in 10 mM NaF. A negative differential score indicates that a hairpin is more likely to be present in 0 mM NaF compared to 10 mM NaF and inversely for a positive score. Our results indicate that, as expected, the first hairpin (P1) is more likely without fluoride whereas the scores do not differ for the second hairpin (P3), which is known to exist in both conditions (Fig. 4c,d). For the third hairpin, the results are dependent on length. For shorter hairpins (stem length ≤7), the scores are about equal between conditions, while longer hairpins (stem length >7) are more likely to form without fluoride. This transition manifests as a hinge-like relationship between the stem size and the differential score, with the transition occurring at hairpins of stem length 7 (Fig. 4e). Interestingly, these results are in perfect agreement with the structures proposed in [23, 74, 75], where the third hairpin is present in both conditions up to stem length 7 and longer stems only form in the absence of a PK (see CT and PT in Fig. 4a, b). Conceptually, this analysis captures the mixed composition of hairpins with varying stem lengths that are found in the sample, from a single base pair to a full 15 nt stem. This zipping/unzipping effect also provides a glimpse into Boltzmann ensemble dynamics.

We then continued our investigation with a larger and more complex motif, namely, the PK in the aptamer domain (see dashed box in Fig. 4b), which we encoded as a binary path of paired and unpaired nucleotides. It was scored in both conditions and for all transcript intermediates to test whether we could reproduce the folding trajectories that were qualitatively inferred in Watters et al. Our results indicate that without fluoride, the PK is present in shorter transcripts (∼65–82 nt) but is destabilized as the transcript elongates until it vanishes when the transcript reaches its mature length (Fig. 4f). On the other hand, the PK is stabilized upon fluoride binding and remains folded as the transcript elongates (Fig. 4g). Also, as expected, scores are generally higher with fluoride, indicating the higher prevalence of the PK. Taken together, our results are in strong agreement with previous studies [23, 74–76]. In summary, we showed that PATTERNA can be used to deduce structural rearrangements in an automated and straightforward manner rather than relying on manual inspection and qualitatively integrating observations from isolated single-nucleotide changes. Furthermore, the capacity to detect PKs—a hallmark of riboswitch structure models—highlights PATTERNA’s potential in aiding genome-scale searches for novel riboswitches [44].

### Hairpin in a haystack: transcriptome-wide search for motifs

Having established PATTERNA’s ability to mine target motifs in small and high-quality data sets, we proceeded to investigate its performance in a more complex transcriptome-wide scenario. To that end, we used PARS data capturing structuromes in a family trio: a father, a mother, and their child [77]. This data set was designed to detect riboSNitches—single-nucleotide variants (SNVs) that result in structural rearrangement within the transcript and can lead to changes in phenotypes [78]—at transcriptome-wide scale. Of particular interest are two riboSNitches, in genes *MRSP21* and *HLA-DRB1*, which were validated by targeted SP using different probes. Allele-specific secondary structures were proposed in Wan et al. This allows us to perform a transcriptome-wide search for these specific structural motifs. We first trained PATTERNA on transcripts filtered for high coverage and sufficient data density and for each subject individually (Additional file 2: Figure S1E–G). We then spiked in synthetic transcripts consisting of the two allelic variants of the *MRPS21* motifs with perfect PARS information, in the child data set. These synthetic transcripts were then used as positive controls to ensure these motifs were properly detected under optimal conditions. We performed a search with no sequence constraints and looked for the target motif’s signature across all transcripts. We used all motif scores to determine the rank of the spike-in regions with perfect information. As expected, spiked motifs ranked first, out of about 2 million scored regions, in both a search for the A or C allele motifs in a pool of 1000 randomly selected transcripts from the child data set, highlighting our ability to readily distinguish them (Additional file 2: Table S2).

**Fig. 5:**
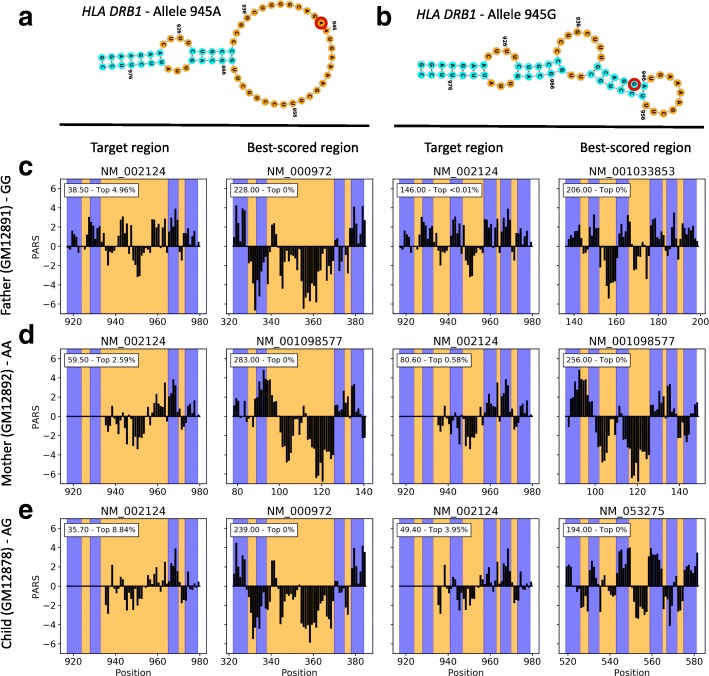
Transcriptome-wide search for the *HLA-DRB1* riboSNitch motif in PARS data. Secondary structure models proposed in Wan et al. for allele variants 945A (**a**) and 945G (**b**) of the *HLA-DRB1* riboSNitch. Red circles highlight the single nucleotide polymorphism. Search results were obtained for the father (homozygote G) (**c**), mother (homozygote A) (**d**), and child (heterozygote) (**e**) data sets. For each riboSNitch variant, PARS traces at both the target location, i.e., the location where the riboSNitch was first reported, and the best-scoring location across tested transcripts are shown. Blue regions indicate helices, i.e., paired nucleotides where positive PARS values are expected, and inversely for orange regions. The inset shows both the score and rank of the scored region relative to all scored regions, where a smaller rank indicates a region is among the best scored ones, with 0% indicating the top scored region

We then searched for both allele-specific secondary structures of the *HLA-DRB1* riboSNitch (Fig. 5a, b) in a pool of 1000 transcripts randomly selected from the original data set and containing both transcripts of interest. The *HLA-DRB1* allele G motif scored highly and significantly better than the A allele for the father, which is homozygote G at that SNV (Fig. 5c). In comparison, compared to the father, the mother (homozygote A) scored poorer for the G allele motif while better for the A allele (Fig. 5d). Note that the structure proposed in Wan et al. for allele A is not strongly supported by the PARS data. Specifically, the proposed motif contains a 32-nt loop, hence negative PARS values are expected in this region. While the 5^′^ end of the loop (nucleotides 935–955) indeed harbors negative values, PARS scores at 956–965 are more consistent with the presence of a helix. This explains why allele A’s motif scored lower than we expected for the mother. The child (heterozygote A/G) did have data more consistent with the G allele motif, yet to a sensibly lesser extent compared to the father (Fig. 5e). For the *MRPS21* motif (Additional file 2: Figure S5A-B), the high data sparsity at the predicted riboSNitch site prevented us from comparing the results across the family (Additional file 2: Figure S5C–E). Nevertheless, the child (heterozygote A/C) provided the best score for the A allele motif and had a profile consistent visually with the proposed motif (Additional file 2: Figure S5E). Moreover, the father (homozygote A) scored best for the A allele and for the mother (homozygote C), there were no differences between alleles (Additional file 2: Figure S5C,D).

Notably, while our results do not entirely support the hypotheses of Wan et al. for the proposed structures of the *MRSP21* and *HLA-DRB1* riboSNitches, we found that the best-scoring regions in each subject and for each tested motif had PARS profiles closely resembling the data signatures expected for these motifs (Fig. 5c–e). This not only suggests that a motif with the sought-after data signature could be present at these locations, which is probable given the relative structural simplicity of the motifs, but also that regions highlighted by PATTERNA are all promising candidates. While PATTERNA does not guarantee the presence of a motif, even for the best-scoring region, it can be used to produce a short list of candidate regions, thereby significantly reducing the search space for motifs of interest, and consequently, simplifying and expediting follow-up validation studies. Moreover, a reduced subset of candidate regions is amenable to a more time-consuming NNTM-based analysis, implying that PATTERNA can be used in conjunction with, rather than as a replacement of, NNTM approaches.

### Assumptions and limitations

A simplifying assumption in our model is that nucleotides assume only two pairing states. Moreover, we assume that all RNAs in a sample share similar structural characteristics, namely that their architectures consist of stretches of paired and unpaired nucleotides, such as helices and loops. This is encapsulated by the HMM, which models a nucleotide’s state as dependent on its adjacent neighbor. To ensure robust estimation and reliable modeling, high data density over transcripts used for training and at scored sites is necessary. This is particularly important because in vivo and transcriptome-wide data sets generally suffer from quality issues due to dramatic variations in transcript coverage and a high prevalence of missing values [79]. We, therefore, recommend applying quality controls prior to training and scoring, similar to those performed in this study. In terms of motif scoring, a target cannot have variable-length gaps in its state sequence, as illustrated in Additional file 2: Figure S6A. Moreover, the downside of the state-sequence representation we use is the inability to discriminate between different motifs whose state paths are identical, for instance, different loop types (Additional file 2: Figure S6B). However, for motifs whose structure is fully known within a contiguous region, e.g., hairpins, sequence information can be used to restrict the search to regions where the sought-after base-pairing pattern is feasible. While this does not guarantee that considered regions contain the motif of interest, sequence constraints greatly reduce the search space and consequently the number of false positives.

## Discussion

### Data-driven structural motif recognition

The recent emergence of high-throughput SP experiments has given rise to novel data-driven applications, which parse transcriptomic data sets to gain insights into the functional role of RNA structures while circumventing the traditional approach of explicitly predicting these underlying structures [26]. This recent paradigm shift warrants the development of new algorithms to glean quantitative information rapidly from transcriptome-wide data sets or large synthetic libraries both at nucleotide resolution and within larger structural contexts. To address this need, we developed PATTERNA, the first pattern recognition algorithm that rapidly detects structural motifs in large-scale SP data sets. It features a machine learning algorithm that learns the statistical properties of SP signals directly from the data, obviating the need for training from reference structures (reference-free) or for applying folding thermodynamics considerations to determine secondary structures explicitly (NNTM-free). PATTERNA utilizes probabilistic modeling to expand the resolution of SP data, which are collected at nucleotide resolution, to that of functional RNA domains, which span at least several nucleotides. This can aid data-driven structure–function studies because often the structural effects of interest are manifested across functional domains and could rarely be reliably deduced from isolated single-nucleotide reactivity changes. Moreover, the latter are often driven by biological or technical noise rather than the underlying biology, rendering the integration of information even more challenging [12]. This point is illustrated well by our analysis of the fluoride riboswitch, where structural rearrangements were previously qualitatively and manually inferred from single-nucleotide reactivity changes [23], but can instead be readily mined when summarizing information over the entire aptamer domain (Fig. 4c, d).

### Probabilistic modeling and interpretation of SP data

PATTERNA’s design is inspired by an analogy between RNA structure and natural language processing, where speech sound units, called phonemes, are the basic building blocks of a word [80]. Similarly, RNA structures (words) can be modeled as a sequence of structural motifs (phonemes) that are themselves composed of a sequence of individual nucleotides (sound waves). In speech recognition, an HMM has traditionally been used to model phonemes as a sequence of transformed sound waves, modeled by a GMM, or more recently by deep neural networks [81, 82]. We adapted this methodology by combining a generative HMM that produces a sequence of pairing states emitting observed SP data, which we also modeled using a mixture of Gaussians. The choice of Gaussians was motivated by our previous observation that applying a log-transformation to SHAPE data induces near-Gaussianity [64].

We showed that our reference-free model performs comparably to a reference-based classifier on SHAPE data, indicating that we recover near-optimal pairing state estimates from SHAPE data alone. Notably, because we generate posteriors that are, by definition, confined between 0 and 1, we are effectively normalizing SP data to an immutable and easily interpretable scale (see Figure 6 in Deng et al. [64]). This is worth noting because there is currently no consensus on how best to normalize SP data [12]. Current approaches heuristically detect outliers to alleviate their impact on normalization [83–87]. Because of their heuristic nature, it is unclear if they (1) are optimal, (2) generalize to diverse experimental contexts, and (3) should be applied to each transcript individually or to an entire transcriptome [12]. In comparison, our probabilistic approach is insensitive to outliers, is remarkably robust to a random initialization of model parameters (Additional file 2: Figure S2), and is broadly applicable to any SP technique and probing condition (Fig. 2 and Additional file 2: Figure S1). This sets a foundation for robust and cross-platform comparative structure analysis.

### PATTERNA rapidly detects motifs in large data set

Using the Weeks benchmark data set, we established that PATTERNA outperforms MFE prediction in detecting motifs, even when predictions are informed by data. This is not too surprising because MFE predictions do not reveal the full complexity of the structure landscape, whereas SP experiments provide an average snapshot of all structures present in a solution. In other words, for a given transcript, a motif might be absent from the MFE structure, while simultaneously present in many other prevalent conformations, hence substantially reducing detection accuracy. This issue is remedied by ensemble-based predictions, as reflected in their superior detection accuracy. However, such predictions are computationally demanding, requiring years at transcriptome-wide scale, and are thus, impractical in the context of recent studies of structuromes [2, 11, 88]. To circumvent this limitation, studies have resorted to folding only a small subset of candidate regions [47, 77, 87, 89] or alternatively to folding smaller fragments [22, 90]. The trade-off in such cases is the potential omission of relevant functional regions or incorrect folding of regions in the absence of their complete structural context [91]. Moreover, to date, we are unaware of any published studies where the Boltzmann ensemble was determined for an entire transcriptome. With PATTERNA, we traded the full details of a RNA secondary structure for its simplified representation as a pairing-state sequence, or its shadow in terms of data signature. This, in turn, greatly reduces computational complexity, bringing down the time requirement to a few hours or days for large transcriptomes, while only moderately affecting detection accuracy compared to ensemble-based performance. Moreover, reducing structures to sequences of states broadens the scope of motifs that PATTERNA can detect. Potential motifs encompass non-nested secondary structures or tertiary pairing interactions, such as the PK in the aptamer domain of the fluoride riboswitch that PATTERNA detected in SHAPE-seq data. This gives PATTERNA a significant advantage over the NNTM paradigm, which cannot consider such motifs efficiently, especially in searches for riboswitches, as they often embed pseudoknotted nucleotides.

We demonstrated that PATTERNA searches effectively for putative functional motifs across an entire transcriptome. Seeking such motifs in a variety of organisms is not a novel endeavor and many methods have been proposed to do so. These methods have in common a search for homologies between the target and transcriptomic regions, but nonetheless, they all consider RNA structure at its primary, secondary and tertiary levels but not through the lens of SP data. SP data, however, delivers supplemental information missed by existing methods, such as the structural rearrangements triggered by changes in the cellular environment. In contrast, PATTERNA offers a solution to find homologies using SP data alone. Furthermore, pattern finding that draws on both sequence structure and SP homologies might prove even more powerful.

## Conclusion

We described the implementation, applications, and limitations of PATTERNA, a pattern recognition machine learning algorithm that rapidly detects RNA structural motifs in large-scale SP data sets. Our results indicate that PATTERNA can effectively detect motifs in various data sets, a task that has not been previously accomplished in a principled and comprehensive manner. Furthermore, PATTERNA can be used to narrow down a set of candidate regions, which can then be used in more careful NNTM analyses, thereby mitigating the computational limitations of NNTM-based methods to enable transcriptome-scale analysis. In motif detection, PATTERNA integrates single-nucleotide information into structural domain knowledge, which has the potential to greatly accelerate the discovery of structural elements of functional importance.

As PATTERNA models the SP signal directly from the data, it is readily applicable to virtually any experimental method that differentiates between paired and unpaired nucleotides (as illustrated in Fig. 2 and Additional file 2: Figure S1). Its flexibility and universality are timely in an era of large-scale data with increasing diversity and complexity, especially as it is becoming clear that many SP methods are complementary rather than redundant [92]. We envision that PATTERNA, as well as related data-driven NNTM approaches [64, 71, 93], will play a critical role in bridging differences within the rapidly growing space of SP methods and their novel downstream applications.

## Methods

### Overview of structure profiling experiments

SP experiments aim at interrogating all RNA structures in a sample at nucleotide resolution and make use of chemical reagents (e.g., SHAPE) or enzymes (e.g., PARS) that are sensitive to the local stereochemistry in the vicinity of a nucleotide [11, 86]. In selective 2 ^′^-hydroxyl acylation analyzed by primer extension (SHAPE) experiments, SHAPE reagents, commonly 1-methyl-7-nitroisatoic anhydride (1M7), N-methyl isatoic anhydride (NMIA), or 2-methylnicotinic acid imidazolide (NAI), form chemical adducts on nucleotides, which interfere with reverse transcription, leading to either reverse transcription terminations or the introduction of mutations. In the newest generation of experiments, these events are assayed by sequencing and a modification rate, called reactivity, is assigned to each nucleotide [60–62, 94]. Briefly, reactivities are obtained by adjusting read counts to account for variations in coverage, yielding two detection rates per nucleotide: one with the reagent (treated sample) and one without it (untreated sample). These rates are combined to estimate the degree of modification at each nucleotide, which is then normalized to ensure the reactivities span the same interval across transcripts and replicates. High and low reactivities are indicative of unpaired and paired nucleotides, respectively (Fig. 1a). Henceforth, a reactivity profile correlates with the underlying assayed structure [29]. Note that it is not uncommon to encounter negative reactivities, which result from technical noise that gives detection rates in the untreated sample exceeding those in the treated one.

In PARS experiments, two nucleases are used: RNAse V1 cleaves double-stranded RNA while RNAse S1 cleaves single-stranded RNA. As with SHAPE, cleavage events are detected by sequencing and S1 and V1 cleavage rates are determined at each nucleotide. The log ratio between V1 and S1 rates is taken at each nucleotide such that a positive/negative score correlates with a paired/unpaired nucleotide (Fig. 1b).

### Overview of PATTERNA

#### Statistical model

There is a detailed description of our model in Additional file 1. Briefly, RNA secondary structure is a base-pairing configuration specified by a list of nucleotides that pair with each other, with remaining nucleotides being unpaired. Since SP data may reveal only a nucleotide’s pairing state but not its pairing partner, we relax the constraint on the pairing partner and represent a secondary structure as a sequence of nucleotide pairing states, where each nucleotide assumes one of two states: paired or unpaired. For example, a hairpin of stem size 4 and loop size 3 is described by the state sequence [1,1,1,1,0,0,0,1,1,1,1], with 0 and 1 representing unpaired and paired bases, respectively. Now, not only do we wish to estimate the probability that a nucleotide assumes a given pairing state, but we also want to incorporate its local structural context into a model [61]. This is because RNA structures often consist of stems and loops, which implies that a nucleotide residing in a loop has a greater probability of being unpaired compared to a nucleotide residing in a stem, irrespective of its observed SP value. In other words, the states of neighboring nucleotides can be informative. We, thus, resorted to Markov chains, which provide short-term contextual memory. As nucleotide pairing states are unknown, a HMM was used to link the unknown underlying structure (i.e., a sequence of hidden and correlated states) to the observed data via initial state, transition and emission probabilities (*π*, *a*, and *b*, respectively). By fitting such a model to the data, one can determine the probability of each hidden nucleotide state [95]. SP data are the observations emitted from our HMM model, which indirectly gives the probability of each hidden state [29, 63, 64].

However, to obtain emission probabilities, we first need to model the observed data as dependent on each pairing state. We use a GMM, a class of flexible models that use multiple weighted Gaussian kernels, which can be combined to reconstruct the shape of any continuous distribution. We use *K* Gaussian components per state, where *K* is user-defined and each Gaussian component is parameterized by its mean (*μ*), variance (*σ*^2^), and weight (*w*) [95]. Furthermore, to consider zeros and missing reactivities, we parameterize them as additional discrete emission probabilities, *υ* and *ϕ*, respectively. The GMM, in conjunction with *υ* and *ϕ*, allows us to estimate emission probabilities at each nucleotide, denoted as *b*, which we then use in our HMM model to obtain posterior pairing state probabilities. This effectively results in a fully integrated GMM-HMM model, which is at the core of PATTERNA. In summary, our framework can be used to determine posterior pairing probabilities at nucleotide resolution directly from SP data, and by extension, the probability of any substructures within that RNA.

#### Training

We trained our model iteratively using the Baum–Welch algorithm, an EM algorithm that utilizes the forward-backward algorithm in the E step [96]. The basic idea that underlies the EM algorithm is that, at each iteration, posterior probabilities of hidden states and of adjacent pairs of states given the data, *γ* and *ξ* respectively, are calculated based on current model parameters *θ*, where *θ*={*a*,*π*,*μ*,*σ*^2^,*w*,*ϕ*,*υ*} (E step). The *γ* and *ξ* posteriors are then used to update the *θ* parameters via the maximization (M step) of a function that derives from the model-based likelihood function $\mathcal {L}$. EM iterations are repeated until there is convergence to a local maximum of $\mathcal {L}$. Default initial values of model parameters are listed in Additional file 1.

#### Extended dot-bracket notation

A secondary RNA structure can be encoded using the dot-bracket notation, where a dot represents an unpaired nucleotide, an open parenthesis represents a nucleotide paired with a nucleotide ahead of it, and a closed parenthesis represents a nucleotide paired with a nucleotide preceding it. For instance, a hairpin of stem size 3 and loop size 4 would be encoded as (((....))). As PATTERNA can take motifs of variable size as input, we added a syntax convention inspired by regular expressions (regex), where a consecutive run of symbols is specified by a symbol followed by the run length in curly brackets. In our example, the hairpin would be encoded as ({3}.{4}){3}. The curly brackets also allow the input of a range of possible run lengths as {*x,y*}, with *x* and *y* the lower and upper bounds of the run length, respectively. For example,.{2,7} would indicate any loops of size 2 to 7.

#### Motif scoring

To score a target motif, we first encoded its secondary structure as the sequence of nucleotide pairing states, which we call the target path. We then considered all possible locations within an RNA where the path may occur. In the absence of sequence constraints, this amounts to scoring the path across all nucleotides within the RNA with no consideration of base-pairing compatibility, similar to a rolling window whose length is set to the target path length. When applying sequence constraints, we restricted the search space to regions where the sequence permits motif formation via Watson–Crick and Wobble base pairings. We scored each region by computing the log ratio of joint probabilities between the target and its opposite path (i.e., the unique path that does not pass through any of the hidden states of the target path) given the trained model (see Additional file 1). Scores were indexed to the nucleotide at the beginning of the target path. Positive scores correspond to regions where the motif is more likely to have occurred relative to its opposite, and inversely for negative scores. Note that these scores can theoretically range from −*∞* to *∞*.

#### Viterbi paths and pairing state probabilities

In addition to motif scoring, our trained model can be used to reconstruct, for a complete transcript, the sequence of binary pairing states that best explains the observed SP data. This sequence, called the Viterbi path, is found by applying the Viterbi algorithm for maximum-likelihood sequence estimation to the GMM-HMM with the emission and transition probabilities determined during the training phase (see Additional file 1). Moreover, pairing-state posterior probabilities, which we denote *γ*, are also generated for each transcript (see Additional file 1). Because our model has binary states at each nucleotide, we do not lose information by retaining the posteriors for the paired state. The resulting *γ*_1_ path is, in essence, the probabilistic (i.e., soft-valued) counterpart of the binary (i.e., hard-valued) Viterbi path.

### Benchmark SHAPE data set

#### Structure prediction

Our benchmark data set was assembled from 21 RNAs with reference secondary structures and SHAPE profiles published by the Weeks lab and summarized in Additional file 2: Table S1 [59, 65, 66]. For each RNA, we predicted MFE secondary structures using RNAprob, a probabilistic method for integrating SP data with the classical NNTM approach to structure prediction, based on the RNAstructure software implementation of the NNTM approach [64, 72]. We predicted structures from both sequence alone and sequence combined with SP constraints, as described previously [64]. In addition, we used GTfold [67] to sample 1000 structures per RNA from the NNTM-based Boltzmann ensemble using both sequence-alone and data-driven partition functions. Note that we refer to both MFE and sub-optimal ensemble structures as NNTM-based predicted structures as they all derive from thermodynamic modeling assumptions. We then encoded both reference and NNTM-based predicted structures as binary vectors of unpaired (0) and paired (1) nucleotides. Next, we trained PATTERNA on both raw and log-transformed SHAPE reactivities to obtain fitted emission distributions and state transitions. Negative SHAPE values were set to zero prior to log-transforming the data and were excluded from the transformation step because PATTERNA internally handles zero SHAPE reactivities using a designated probability parameter (see Additional file 1). We trained our model using ten Gaussian components per pairing state.

To benchmark PATTERNA predictions, we used the trained GMM-HMM to reconstruct the sequence of binary pairing states that best explains the observed reactivities. This sequence, called the Viterbi path, was found for each RNA by applying the Viterbi algorithm for maximum-likelihood sequence estimation to the GMM-HMM with the emission and transition probabilities that were determined in the training phase (see Additional file 1). Additionally, for each nucleotide *t* (1≤*t*≤*T*), we computed the posterior probability that it is paired (i.e., in state 1) given the data, which we denote *γ*_1,*t*_. Before computing an accuracy measure, we concatenated all the RNAs such that each method is represented by a single vector of length *L*. As the analysis included both binary (i.e., reference, MFE, and Viterbi structures) and continuous vectors (i.e., *γ* path), we determined prediction performances using the *ℓ*_1_-norm between reference and predicted structures:

1 $$ \begin{aligned} \ell_{1} &= \sum_{l=1}^{L} |y_{l}-\hat{y_{l}}|,~\text{with} \\ y &= \text{reference structure} \\ \hat{y} &= \text{predicted structure}. \end{aligned}  $$

An advantage of the *ℓ*_1_-norm is that it is equivalent, for two binary vectors, to the Hamming distance, defined as $\sum _{\forall l} y_{l} \oplus \hat {y_{l}}$, and we can compute the prediction accuracy as

2 $$ \text{Accuracy} = 1 - \frac{\ell_{1}}{L}.  $$

Finally, we also considered structures predicted by a simple, yet trained, classifier, which thresholds reactivities into unpaired (0) and paired (1) states using a reference-based optimized cutoff. Both raw and log-transformed data were classified, and the threshold was set to the value that minimizes the *ℓ*_1_-norm between the resulting binary vector and the reference structure (Additional file 2: Figure S7). Note that for missing reactivities, we assigned a classification score of 0.5, meaning there is an equal probability of being paired or unpaired. Moreover, for log-transformed data, the original zero and negative SHAPE values, which cannot be transformed, were assigned to paired nucleotides.

#### Fragmentation analysis

To mimic transcriptome-wide motif searches that use NNTM-based predictions [22, 90], we partitioned RNA sequences and SHAPE profiles into non-overlapping 100-nt long fragments. After partitioning, if less than 100 nt remained at the 3^′^ end, we appended them to the previous adjacent 100-nt fragment to ensure that no fragment was smaller than 100 nt. For RNAs shorter than 100 nt, we used a single fragment consisting of the complete RNA. We then predicted MFE and suboptimal ensemble structures for each fragment independently, following the same steps as for non-fragmented RNAs. Finally, we encoded each folded fragment into unpaired (0) and paired (1) nucleotides and assembled fragment-based structures into full-length RNAs, which we then processed identically to unfragmented RNAs.

#### ROC analysis of motif predictions

We tested the detection accuracy of NNTM-based methods and PATTERNA for three motif types: loops, hairpins, and hairpin-right bulge composites. We specifically searched for the following motifs encoded in the extended dot-brackets notation (see “Extended dot-bracket notation” section):

- Loops:
-   (.{3,10})
- Hairpins:
-   ({2,20}.{3,10}){2,20}
- Hairpin-right bulges:
-   ({1,10}.{3,10}){1,5}.{1,5}){1,15}

Sequence constraints on paired nucleotides were applied when searching for hairpins and hairpin-bulges but not for loops. To assess performance, we considered all regions scored by PATTERNA and established the presence or absence of the motif’s binary state path based on the known reference structures. For ensemble samples, we verified the presence or absence of the motif at each nucleotide and in each sampled structure and recorded the frequency at which the motif was observed in the sample. Similarly, for the MFE structure, we recorded whether the motif was present or absent at each nucleotide, resulting in a binary vector. For each motif, we obtained from the reference structure a ground truth binary vector, *y*, summarizing the presence or absence of the motif at each scored location. Score vectors obtained for each of the benchmarked methods ($\hat {y}$) were thresholded and compared to *y* via ROC analysis using the SCIKIT-LEARN Python package [97].

### Fluoride riboswitch analysis

We used in vitro SHAPE-seq data for the *B. cereus* fluoride riboswitch publicly available in the RNA Mapping Database (Additional file 2: Table S3) [23]. This data set consists of three replicates of fluoride riboswitch co-transcripts. Each cotranscript corresponds to a sequence position in which transcript elongation was arrested. Cotranscripts were SHAPE-profiled in the absence and presence of 10mM NaF. A set of 2272 transcripts was prepared by combining all probed fluoride riboswitch transcripts across replicates and conditions. We trimmed the last 10 nt at the 3^′^ end of the transcripts to remove RNA polymerase footprints that block the SHAPE reagent. To train our model, we excluded all transcripts that contained over 10% of missing values, i.e., their SHAPE densities fell below 90%, resulting in a training set of 230 transcripts. We used ten Gaussian components in our GMM, which were initialized in the default way. We then scored hairpins ranging from stem sizes of 1 to 20 nt and loop sizes of 4 to 6 nt on the full-length transcript (90 nt) for each replicate independently. To enrich our results for hairpins, we applied sequence constraints on paired nucleotides, effectively ensuring that we were scoring only regions that can form hairpins. We computed the final scores by averaging over replicate scores.

We considered that the PK in the aptamer domain consisted of 45 nt that spanned the region from nucleotide 5 to nucleotide 49. We encoded the motif using the following dot-bracket representation:.({16}.{6}({3}.{4}){4}.(.){8}, where numbers in curly brackets indicate repeats of the previous characters (see “Extended dot-bracket notation” section in “Methods” section). Note that we accommodated nucleotides involved in long-range interactions by considering them as being in a paired state, as they are protected from SHAPE modification and we did not use sequence constraints. We scored the PK at any possible starting nucleotide, in both experimental conditions and for transcript lengths ranging from 30 to 100 nt, i.e., all available intermediate transcripts. To remove artifacts due to the polymerase footprint, we trimmed an additional 5 nt, resulting in a total of 15 nt trimmed at the 3^′^ end of each transcript. We computed the final scores by averaging over replicate scores.

### Motif searches in transcriptome-wide PARS data

We used human in vitro transcriptome-wide PARS data measured in a family trio consisting of a father (GM12891), mother (GM12892), and their child (GM12878) (Additional file 2: Table S3) [77]. We retrieved V1 and S1 read counts for these subjects and computed PARS scores as described in Wan et al.:

3 $$ \text{PARS}_{t} = \log_{2}(\text{V1}_{t} + 5) - \log_{2}(\text{S1}_{t} + 5).  $$

Like the quality control applied in Wan et al., we excluded all transcripts with combined coverage across the V1 and S1 channels lower than 1 read per nucleotide and excluding 100 nt at the 3^′^ end. For instance, we required that a transcript of length 500 was covered by at least 400 sequencing reads mapped from either the V1 or S1 channels. To ensure the accurate estimation of transition probabilities during training, we further excluded transcripts with PARS densities lower than 50%. Subjects’ training sets initially consisted of 2737, 2506, or 2512 highly covered transcripts and after filtering for sufficient density, we kept 2027, 1935, and 1983 transcripts for the father, mother, and child, respectively. We trained PATTERNA on each subject separately because there are no guarantees that technical and biological variations are shared across subjects. Furthermore, to ensure consistency while training across subjects, we initialized the GMM’s Gaussian components at unit variance with identical weights across components and symmetrical means at {−1,−2,−3} and {1,2,3} for unpaired and paired states, respectively.

We investigated the presence of the two allelic versions of the MRPS21 (291A>C) and HLA-DRB1 (945G>A) riboSNitches, since secondary structure models were proposed in Wan et al. These structures translate to the following in dot-bracket notation:

- MRPS21 A (NM_018997, start 268):
-   .(((((((.......(((((.......))))).......))))))).
- MRPS21 C (NM_018997, start 275):
-   .(((((....(((((.((((.((........)).)))).)))))..)))))...
- HLA-DRB1 G (NM_002124, start 917):
-   (((((((....(((((........((((((..........))))))..)))))...)))))))
- HLA-DRB1 A (NM_002124, start 917):
-   (((((((....(((((................................)))))...)))))))

As a positive control, for each riboSNitch, we spiked into the child’s test data set two synthetic transcripts consisting of the two allelic variants of the *MRPS21* motif with perfect PARS information padded with 20 zeros on both sides. We defined perfect information as unpaired and paired nucleotides with a constant PARS value equal to the 2.5% (PARS=−2.70) and 97.5% (PARS=2.55) percentiles, respectively. Percentiles were computed from 1,000,000 randomly sampled data points. We then conducted transcriptome-wide searches for these riboSNitches in each subject using 1000 transcripts randomly selected from the pool of highly covered transcripts. As we aimed at establishing motif detection accuracy in the broadest possible context, we did not apply sequence constraints when scoring motifs. To compare scored regions across subjects, scores were first sorted in descending order, that is, from more to less likely motifs, and the rank of the target motif was used to compute a simple statistical metric defined as the rank divided by the total number of scored regions. For instance, if a target motif score ranked tenth out of 100 tested motifs, the resulting metric would be 10/100=10*%*. We used the average across ranks when a motif’s score was not unique.

## Additional files

Additional file 1 Supplementary Methods. Detailed mathematical description of the methods underlying patteRNA. (PDF 233 kb)

Additional file 2 Supplementary Figures and Tables. (PDF 7600 kb)

Additional file 3 Reviewer reports and author’s response to reviewers. (DOCX 21 kb)

## Abbreviations

AUC: Area under the curve
EM: Expectation-maximization
GMM: Gaussian mixture model
GMM-HMM: Gaussian mixture model-hidden Markov model
HMM: Hidden Markov model
MFE: Minimum free energy
NNTM: Nearest-neighbor thermodynamic model
PK: Pseudoknot
ROC: Receiver operating characteristic
SP: Structure profiling
SNV: Single-nucleotide variant
