# Supplementary material for: PATTERNA: transcriptome-wide search for functional RNA elements via structural data signatures
Source: Genome Biol. 2018 Mar 1;19:28. doi: 10.1186/s13059-018-1399-z (PMC5833111; doi:10.1186/s13059-018-1399-z)
Supplement: Supplementary file 1 — Supplementary Methods. Detailed mathematical description of the methods underlying patteRNA. (PDF 233 kb) [file 13059_2018_1399_MOESM1_ESM.pdf]

Supplementary Method for "patteRNA: transcriptome-wide search for functional RNA elements via structural data signatures"

Mirko Ledda and Sharon Aviran

Department of Biomedical Engineering and Genome Center, University of California at Davis, USA.

Introduction

Table 1: Glossary of symbols

Symbol	Definition (dimensions)
N	Number of possible hidden states in the HMM
T	Length of the RNA
Q	Number of RNAs in the dataset
K	Number of Gaussian components in the mixture PDF
θ	Complete set of parameters
ω	Hidden states in the HMM ($N \times T$)
y	Observed reactivities ($T \times 1$)
π	Initial state probabilities ($N \times 1$)
a	Transition probabilities ($N \times N$)
b	Emission probabilities ($N \times T$)
w	Mixture coefficients ($N \times K$)
μ	Means ($N \times K$)
σ^2	Variances ($N \times K$)
ϕ	Emission probabilities for missing observations ($N \times 1$)
υ	Emission probabilities for observed zeros ($N \times 1$)
α	Forward probabilities ($N \times T$)
β	Backward probabilities ($N \times T$)
γ	State occupation probabilities ($N \times K \times T$)
ξ	Joint transition event probabilities ($N \times N \times T - 1$)
$\mathcal{L}$	Likelihood

Model overview

The general objective of our framework is to infer the probability of a target RNA structure motif across an entire dataset, given observed SP data. We first consider a simplified example where we restrict search to a single motif and a single transcript and assume knowledge of the transcript's secondary structure. The presence or absence of the motif can then be established by directly comparing it to the structure of the RNA. Simply put, we can encode both the target motif and the transcript's structure in terms of numerical base pairing states, say 1 for paired and 0 for unpaired, and scan the transcript for the motif. In practice, a transcript's structure is unknown and not directly observable, in other words, the base pairing states are hidden. What we observe instead is a range of continuous values $y = \{y_1, y_2, \ldots, y_T\}$ emitted from an underlying sequence of hidden pairing states $\omega = \{\omega_1, \omega_2, \ldots, \omega_T\}$, with T the transcript's length. It is trivial to see that if we were given perfect data, for instance an hypothetical experiment generating 0/1 for unpaired/paired nucleotides, we could have determined each hidden state perfectly from this data. In summary, our goal is to infer the sequence of hidden pairing states that is most likely to have generated the observed data. Moreover, we want to make inferences for each nucleotide in its structural context, or in other words, taking into consideration (i.e. remembering) its neighbors' pairing states. This type of problem is typically approached using Hidden Markov Models (HMMs), a probabilistic framework to estimate a sequence of hidden states while simultaneously introducing finite memory in the system.
To infer the probability of a sequence of hidden states ω we need to know two fundamental quantities: transition and emission probabilities. A transition probability is the probability that a nucleotide $t+1$ is in pairing state j given that the preceding nucleotide t is in state i. Assuming this probability remains constant across the entire dataset, we denote it as

$$a_{i,j} = \Pr(\omega_{t+1} = j | \omega_t = i), \tag{1}$$

where for each i, transitions sum to 1 over states j, s.t. $\sum_j a_{i,j} = 1 \ \forall i$.
An emission probability is the likelihood of observing a value y_t given that nucleotide t is in pairing state $\omega_t = i$ which we write as

$$b_{i,t} = \Pr(y_t | \omega_t = i). \tag{2}$$

We wish to infer the posterior probabilities of the hidden states given the observed data y, however at this stage our model parameters - a and b - are unknown and we must first train them. What we need is parameters, denoted by θ, that best explain the data we observed. In other words, we want to find the maximum *a posteriori* (MAP) estimates of our parameters given the observed data which we write as

$$\arg\max_\theta \Pr(\theta | y) = \frac{\Pr(y|\theta)\Pr(\theta)}{\Pr(y)}. \tag{3}$$

We impose no prior beliefs on model parameters θ and thus we assume that they are uniformly distributed implying that $\Pr(\theta)$ are constants. Additionally, the probability of the data $\Pr(y)$ is not dependent on θ in which case we can simplify the posteriors of our model parameters to

$$\Pr(\theta|y) \propto \Pr(y|\theta). \tag{4}$$

It should now be clear that by maximizing the likelihood function $\mathcal{L} = \Pr(y|\theta)$ we are in effect maximizing the posterior probabilities $\Pr(\theta|y)$. The maximization of the likelihood function $\mathcal{L}$ for HMMs has been solved decades ago [1, 2] and makes use of an expectation-maximization (EM) algorithm. EM is an iterative hill-climbing algorithm that is especially suited for maximizing likelihood functions for models that involve hidden information of discrete nature and a continuous parameter space. Each iteration comprises of two steps: E-step, where hidden states are being imputed under the θ estimates at that iteration, and M-step, where model parameters are updated by maximizing a likelihood-derived function. The EM-steps are repeated until convergence of the model likelihood $\mathcal{L}$.

Model parameters

Before describing the specifics of our model's training, we must first define the exact nature of the θ parameters. As mentioned earlier, transition probabilities a are part of the θ parameters but so far we did not mention the special case occurring when we begin a new sequence. At the initial nucleotide $t = 1$, no prior transition could have possibly happened and therefore we must start in some hidden state i based on some initial probability derived from the observed data that we denote π_i and define as

$$\pi_i = \Pr(\omega_1 = i|y_1), \ \texttt{with} \ 1 \leq i \leq N. \tag{5}$$

We next need to estimate emission probabilities but because profiling experiments generate continuous data, we have no means to produce b in a direct manner. What we need instead is a model that fits the observed data and from which we can generate an emission probability $\Pr(y_t|\omega_t)$ for each observed data point. For transcripts with known secondary structures, $\Pr(y_t|\omega_t)$ is obtained by categorizing the observed data based on nucleotide's pairing states, the hidden states ω in our HMM, and by fitting each resulting data distribution independently [3, 4]. For each state, we can now find a probability density function (PDF) that appropriately fits the distribution. In practice, however, we do not know the structure of the transcript nor the PDFs associated with each hidden state and we therefore demand a dynamic model that can fit the data adaptively.

One such class of models is called a Mixture Model (MM) and makes use of multiple PDF functions derived from a common kernel function that, when aggregated, can fit the shape of any distributions. The most widely studied

MM uses a Gaussian kernel, denoted $\mathcal{N}$, and is known as the Gaussian Mixture Model (GMM). Moreover, the integration of a GMM within an HMM, known as a GMM-HMM, is a very well studied subject and proved to be very successful, most notably for speech and hand-writing recognition. The basic idea behind a GMM is that we can approximate a data distribution by a finite sum of K Gaussian distributions weighted using mixture coefficients w. A single Gaussian distribution is parametrized by a mean μ and a variance σ^2. For the entire GMM model we have $K \times N$ Gaussian functions, one for each mixture component K and each hidden state N, and therefore we need to estimate $K \times N$ sets of parameters $\{\mu, \sigma^2, w\}$. Formally, we write a GMM as

$$b_{i,t} = \Pr(y_t|\omega_t = i) = \sum_{k=1}^{K} w_{i,k}\ \mathcal{N}(y_t; \mu_{i,k}, \sigma^2_{i,k})\ , \qquad 1 \leq k \leq K, 1 \leq t \leq T$$

with the constraints

$$\sum_{k=1}^{K} w_{i,k} = 1$$

$$w_{i,k} \geq 0\ , \qquad 1 \leq k \leq K$$

$$\int_{-\infty}^{\infty} b_{i,y} \mathtt{d}y = 1$$

$$\texttt{with } 1 \leq i \leq N \qquad (6)$$

Using equation (6) we can compute emission probabilities for any continuous observation. However, missing reactivities are common in sequencing-based profiling experiments and rejecting an entire profile because of missing observations, denoted $\varnothing$, would result in significant data loss. Similarly, zero reactivities are often encountered either naturally for PARS experiments or when negative reactivities are set to 0, as is often the case for SHAPE, DMS, or similar chemical modification experiments. A multitude of zeros yields a large discrete spike in the PDF and therefore, they are better interpreted independently.

Moreover, reactivities can be log-transformed prior to training the model or computing target motif scores, as this has been previously shown to induce Gaussianity [4]. Missing values and zero reactivities are accommodated by introducing new vectors [5], denoted ϕ for missing values and υ for zero reactivities. Since emissions are modeled as state-dependent, we classify these special values for each state as

$$\phi_i = \Pr(y_t = \varnothing, \omega_t = i)$$

$$\upsilon_i = \Pr(y_t = 0, \omega_t = i) \tag{7}$$

We now have three possible options to compute emission probabilities but by definition, those must sum to 1 across the set of all possible observations. This directly implies that the area under the PDF, $\int_{-\infty}^{\infty} b_{i,y} \mathtt{d}y$, must be smaller than 1 if either $\phi_i > 0$ or $\upsilon_i > 0$. We can therefore generalize equation (6) to accommodate these new discrete probability vectors by scaling the area under the PDF and write our entire GMM model as

$$b_{i,k,t} = \begin{cases} \phi_i \; w_k & ,\texttt{if } \; y_t = \varnothing \\ \upsilon_i \; w_k & ,\texttt{if } \; y_t = 0 \\ (1 - \phi_i - \upsilon_i) \; w_k \; \mathcal{N}(y_t; \mu_{i,k}, \sigma^2_{i,k}) & ,\texttt{otherwise} \end{cases}$$

$$b_{i,t} = \sum_{k=1}^{K} b_{i,k,t}$$

$$\texttt{for } \; 1 \le i \le N, \quad 1 \le t \le T, \quad 1 \le k \le K \tag{8}$$

To simplify and improve the fitting of emission PDFs with the mixture function, reactivities can be log-transformed prior to training the model or computing target motif scores, as this has been previously shown to induce Gaussianity [4]. However, zero reactivities, which are highly prevalent in structure profiling datasets, cannot be log-transformed. To properly account for these, they are not discarded but rather modeled using the υ vector as described in equation (8), such that υ models zeros observed in pre-transformed input profiles.

To summarize, we need to train the initial state probabilities π, the transition probabilities a, the parameters of our GMM $\{\mu, \sigma^2, w\}$ and the emission probabilities for missing and zero observations $\{\phi, \upsilon\}$. We can therefore write the entire set of parameters of our GMM-HMM model as

$$\theta = \{a, \pi, \mu, \sigma^2, w, \phi, \upsilon\}$$

Model training using Expectation-Maximization

To train our GMM-HMM model, we need to find the set of parameters θ that best explain the observed data as described in equation (3). These parameters are found using a conventional Expectation-Maximization (EM) algorithm that uses the forward-backward algorithm, an approach most commonly referred to

as the Baum-Welch algorithm [1]. Briefly, the model is trained iteratively. At each iteration, probabilities of hidden states are computed for each nucleotide, given current θ parameter values, in what is called the E-step. These probabilities are subsequently used to update the model parameters, in what is called the M-step. Updated parameter values are those that maximize a function that derives from the model's likelihood function $\mathcal{L}$. E and M steps are then repeated until convergence of the model's likelihood within a defined tolerance threshold.

E Step

During the E step, we need to infer, for each nucleotide, the posterior marginals of the hidden states given current θ parameter estimates and the entire sequence of observed data, which we denote

$$\gamma_{i,t} = \Pr(\omega_t = i|\theta, y).$$

We also wish to infer the joint probability that nucleotide t is in state i and that its adjacent nucleotide $t+1$ is in state j, which we write

$$\xi_{i,j,t} = \Pr(\omega_t = i, \omega_{t+1} = j|\theta, y).$$

These probabilities are obtained in two passes using a dynamic programming framework called the forward-backward algorithm. During the forward-pass, we compute the probability of ending in state i at nucleotide t given the first t observed data points, or $\alpha_{i,t} = \Pr(\omega_t = i|\theta, y_{1:t})$. In the backward-pass, we use $\omega_t = i$ as the starting point and compute the probability of the remaining observed data, or $\beta_{i,t} = \Pr(y_{t+1:T}|\theta, \omega_t = i)$. We write the forward-backward recursions as follows

$$\begin{aligned}
&c_t = \frac{1}{\sum_{i=1}^{N} \alpha_{i,t}} \ , && 1 \le t \le T \\
&\hat{\alpha}_{i,1} = c_1 \pi_i b_{i,1} \\
&\hat{\alpha}_{j,t+1} = c_{t+1} b_{j,t+1} \sum_{i=1}^{N} \hat{\alpha}_{i,t} a_{i,j} \ , && 2 \le t \le T-1 \\
&\hat{\alpha}_{i,T} = c_T \ \alpha_{i,T} \\
&\hat{\beta}_{i,t} = c_t \sum_{j=1}^{N} a_{i,j} b_{j,t+1} \hat{\beta}_{j,t+1} \ , && t = [T-1, T-2, \ldots, 1] \\
&\hat{\beta}_{i,T} = c_T
\end{aligned} \tag{9}$$

Note the introduction of a scaling constant c_t after each nucleotide t to compute scaled forward $\hat{\alpha}$ and backward $\hat{\beta}$ probabilities [2, 6]. This scaling is necessary as otherwise unscaled α and β would become very small as recursions progress and would result in numerical overflow. Moreover, the scaling constant c normalizes the likelihood contributed by a single nucleotide t, or $\Pr(y_t|\theta) = 1/c_t$. It therefore follows that multiplying $\Pr(y_t|\theta)$ across all nucleotides $\in T$ will provide us the likelihood $\mathcal{L}$ of the entire model [2] which we write in the log domain as

$$\log \mathcal{L} = \log[\Pr(y|\theta)] \propto \log \left[\frac{1}{\prod_{t=1}^{T} c_t} \right] = -\sum_{t=1}^{T} \log c_t \tag{10}$$

We can now combine $\hat{\alpha}$ and $\hat{\beta}$ to estimate posterior state probabilities γ and the joint state event probabilities ξ. However, we wish to do so for all transcripts in the dataset simultaneously and we therefore need to take into consideration each transcript's likelihood. Denoting the number of transcripts in the dataset by Q, we have for a transcript q a sequence of observed data $y^{(q)}$. We can now compute ξ for each transcript as follow

$$\xi_{i,j,t}^{(q)} = \hat{\alpha}_{i,t}^{(q)} a_{i,j} b_{j,t+1}^{(q)} \hat{\beta}_{j,t+1}^{(q)}\ , \quad 1 \leq t \leq T-1 \tag{11}$$

For γ, we need to account for both transcript's likelihood and individual contributions from Gaussian mixture components. As denoted in (10), the scaling constants c represent the contribution of each nucleotide to a transcript's likelihood and we can therefore use this property to scale γ. On the other hand, the contribution of a Gaussian mixture component to the emission probability b is the proportion of the probability emitted from that component compared to all components combined, or

$$\frac{b_{i,k,t}^{(q)}}{\sum_{p=1}^{K} b_{i,p,t}^{(q)}}$$

Using these two properties we can write γ as

$$\gamma_{i,k,t}^{(q)} = \frac{1}{c_t^{(q)}} \left[\hat{\alpha}_{i,t}^{(q)} \hat{\beta}_{i,t+1}^{(q)} \right] \left[\frac{b_{i,k,t}^{(q)}}{\sum_{p=1}^{K} b_{i,p,t}^{(q)}} \right], \qquad 1 \leq t \leq T-1$$

$$\gamma_{i,k,T}^{(q)} = \hat{\alpha}_{i,T}^{(q)} \left[\frac{b_{i,k,T}^{(q)}}{\sum_{p=1}^{K} b_{i,p,T}^{(q)}} \right] \tag{12}$$

$$\texttt{with}\ 1 \leq i \leq N, 1 \leq j \leq N, \\ 1 \leq k \leq K, 1 \leq q \leq Q$$

The likelihood of the GMM-HMM model for multiple transcripts is simply the product of the likelihood of each individual transcipt's model. In the log domain this is computed as

$$\log \mathcal{L} = \sum_{q=1}^{Q} \log \mathcal{L}^{(q)} \tag{13}$$

M Step

During the M-step, we want to update the model parameters $\theta = \{a, \pi, \mu, \sigma^2, w, \phi, v\}$ to maximize a likelihood-derived function, or in other words, find updated parameters that best explain the data we imputed. For our model, this maximization is performed by re-evaluating each parameter using the expected values of the pairing state probabilities of each nucleotide and the transition probabilities, γ and ξ, obtained during the E-step. Intuitively, γ and ξ are simply our current best guesses regarding the hidden states of our model and it naturally follows that we want to use these to update our model parameters. Moreover, because we generated scaled ξ and γ in equations (11) and (12), these can be thought as pseudo-counts of our hidden states and can be used directly to obtain event frequencies. For example, let us consider π_i, the probability that hidden state i generated the observed value at nucleotide $t = 1$. This probability is obtained by counting the number of times we were in state i at nucleotide $t = 1$, across transcripts and Gaussian mixture components, and dividing by the number of times we encountered nucleotide $t = 1$. In other words, we are calculating the expected frequency of state i at nucleotide $t = 1$ across the entire dataset. Also, as this frequency is not dependent on any particular Gaussian component K , we can first simplify γ as

$$\begin{aligned} \overset{\diamond}{\gamma}{}^{(q)}_{i,t} &= \sum_{k=1}^{K} \gamma^{(q)}_{i,k,t} \\ \texttt{with } & 1 \le i \le N,\ 1 \le t \le T,\ 1 \le q \le Q \end{aligned} \tag{14}$$

This summation of γ over Gaussian components K allows us to simplify the expression of π as

$$\bar{\pi}_i = \frac{\sum_{q=1}^{Q} \overset{\diamond}{\gamma}{}^{(q)}_{i,1}}{\sum_{i=1}^{N} \sum_{q=1}^{Q} \overset{\diamond}{\gamma}{}^{(q)}_{i,1}} \ , \quad 1 \le i \le N \tag{15}$$

Similarly, we can update the transition probabilities a by counting the number of transitions from state i to state j, which is given by ξ, and divide by the expected number of times we were in state i, which we write

$$\bar{a}_{i,j} = \frac{\sum_{q=1}^{Q} \sum_{t=1}^{T^{(q)}-1} \xi_{i,j,t}^{(q)}}{\sum_{q=1}^{Q} \sum_{t=1}^{T^{(q)}-1} \overset{\diamond}{\gamma}{}_{i,t}^{(q)}} \tag{16}$$

$$\texttt{with } 1 \leq i \leq N, 1 \leq j \leq N$$

To estimate ϕ, we need to know the probability of observing a missing value given we are in state i. We can get this probability by counting the number of times we were in state i when nucleotide t emitted a missing value divided by the total occurrences of all possible states, or

$$\begin{aligned} \bar{\phi}_i &= \frac{\sum_{q=1}^{Q} \sum_{t=1}^{T} \overset{*}{\gamma}{}_{i,t}^{(q)}}{\sum_{i=1}^{N} \sum_{q=1}^{Q} \sum_{t=1}^{T} \overset{\diamond}{\gamma}{}_{i,t}^{(q)}} \\ \texttt{with} & \\ \overset{*}{\gamma}{}_{i,t}^{(q)} &= \begin{cases} \overset{\diamond}{\gamma}{}_{i,t}^{(q)} & , \texttt{ if } y_t^{(q)} = \varnothing \\ 0 & , \texttt{ if } y_t^{(q)} \neq \varnothing \end{cases} \\ 1 \leq i &\leq N, 1 \leq t \leq T \end{aligned} \tag{17}$$

In the same fashion, we can write the update formula for υ, the probability of a zero observation, as

$$\begin{aligned} \bar{\upsilon}_i &= \frac{\sum_{q=1}^{Q} \sum_{t=1}^{T} \overset{*}{\gamma}{}_{i,t}^{(q)}}{\sum_{i=1}^{N} \sum_{q=1}^{Q} \sum_{t=1}^{T} \overset{\diamond}{\gamma}{}_{i,t}^{(q)}} \\ \texttt{with} & \\ \overset{*}{\gamma}{}_{i,t}^{(q)} &= \begin{cases} \overset{\diamond}{\gamma}{}_{i,t}^{(q)} & , \texttt{ if } y_t^{(q)} = 0 \\ 0 & , \texttt{ if } y_t^{(q)} \neq 0 \end{cases} \\ 1 \leq i &\leq N, 1 \leq t \leq T \end{aligned} \tag{18}$$

Lastly, we need to update the parameters of our GMM $\{\mu, \sigma^2, w\}$. We can use the same strategy we applied so far, i.e. using expected pseudo-counts, but this time we need to consider only nucleotides that did not emit a missing or zero observation as those are accounted for in ϕ and υ. We write the update formula as (note that Gaussian component specific γ are used)

with

$$\overset{*}{\gamma}{}^{(q)}_{i,k,t} = \begin{cases} \gamma^{(q)}_{i,k,t} & , \texttt{ if } y^{(q)}_t \notin \{0, \varnothing\} \\ 0 & , \texttt{ if } y^{(q)}_t \in \{0, \varnothing\} \end{cases}$$

$$1 \leq i \leq N, 1 \leq k \leq K$$

we get

$$\bar{\mu}_{i,k} = \frac{\sum_{q=1}^{Q} \sum_{t=1}^{T} \overset{*}{\gamma}{}^{(q)}_{i,k,t} y^{(q)}_t}{\sum_{q=1}^{Q} \sum_{t=1}^{T} \overset{*}{\gamma}{}^{(q)}_{i,k,t}} \tag{19}$$

$$\bar{\sigma}_{i,k} = \frac{\sum_{q=1}^{Q} \sum_{t=1}^{T} \overset{*}{\gamma}{}^{(q)}_{i,k,t} (y^{(q)}_t - \mu^{(q)}_{i,k})^2}{\sum_{q=1}^{Q} \sum_{t=1}^{T} \overset{*}{\gamma}{}^{(q)}_{i,k,t}}$$

$$\bar{w}_{i,k} = \frac{\sum_{q=1}^{Q} \sum_{t=1}^{T} \overset{*}{\gamma}{}^{(q)}_{i,k,t}}{\sum_{q=1}^{Q} \sum_{k=1}^{K} \sum_{t=1}^{T} \overset{*}{\gamma}{}^{(q)}_{i,k,t}}$$

Convergence criterion

The GMM-HMM model is fitted iteratively to the data using the EM algorithm until the difference between two consecutive model's log likelihoods become smaller than a user-defined tolerance, we call epsilon. We write the convergence criterion as

$$\log \mathcal{L}_i - \log \mathcal{L}_{i-1} \geq |\epsilon \log \mathcal{L}_i| \text{ }, \forall i \geq 5 \tag{20}$$

Note that at least 5 iterations are performed before we check for convergence of the model.

Parameters initialization and input arguments

Our algorithm is parametrized by a set of immutable user-defined input arguments and θ parameters for the GMM-HMM model. The default values of relevant user-defined arguments are

- Number of Gaussian components per states (K): 10
- Convergence criterion (ϵ): 10^{-4}
- Maximum number of EM iterations: 100
- The initial value of the parameters before training are listed below for $N = 2$ states denoted by [`unpaired`, `paired`] and 1 Gaussian component per state:

- Transition probabilities (a):

$$\begin{bmatrix} 0.5 & 0.5 \\ 0.5 & 0.5 \end{bmatrix}$$

- Initial probability (π): [0.5, 0.5]
- Gaussian means (μ):
- Gaussian means are initialized using a random value drawn from a uniform distribution U parameterized by the min, max or median value of $\widetilde{y}$, where y is all input training observations.
  - PARS $\sim \Big[U\{\min(y), \widetilde{y}\}, U\{\widetilde{y}, \max(y)\}\Big]$
  - SHAPE $\sim \Big[U\{\widetilde{y}, \max(y)\}, U\{\min(y), \widetilde{y}\}\Big]$
- Gaussian variances (σ^2): $\Big[\widehat{\text{Var}}(y), \widehat{\text{Var}}(y)\Big]$
- Gaussian weights (w): $\Big[\frac{1}{K}, \frac{1}{K}\Big]$
- NaN observations (ϕ): [0.5, 0.5]
- Zero observations (υ): [0.5, 0.5]

The same GMM parameters $\{\mu, \sigma^2, w\}$ are repeated for larger number of Gaussian components K.

Motif scoring

To score a motif we consider the joint probability of the observations y and the target state-sequence (i.e. the motif's path) given the trained model. For example, consider a hairpin of stem size 4 and loop size 3, starting at nucleotide m and of length $n = 4 + 3 + 4 = 11$. Such motif will produce the following state path $z = \{1, 1, 1, 1, 0, 0, 0, 1, 1, 1, 1\}$, with 0 and 1 representing unpaired and paired nucleotides, respectively. We can write the joint probability of the data and z, denoted $\Pr(y, z|\theta)$, as:

$$\begin{aligned} \Pr(y, z|\theta) = &\Pr(y_1, \ldots, y_{m-1}|\theta)\cdot \\ &\Pr(y_m, \omega_m = z_m|\theta)\cdot \\ &\Pr(y_{m+1}, \ldots, y_{m+n}|\omega_{m+1} = z_{m+1}, \ldots, \omega_{m+n} = z_{m+n}, \theta)\cdot \\ &\Pr(\omega_m = z_m, \ldots, \omega_{m+n} = z_{m+n}|\theta)\cdot \\ &\Pr(y_{m+n+1}, \ldots, y_t|\omega_{m+n} = z_{m+n}, \theta) \end{aligned} \tag{21}$$

While this expression seems complex at first glance, it is simply a breakdown of probabilistic components involved in the joint probability calculation. In words,

this amounts to calculating the joint probability of: 1-2) all observations from the beginning of the transcript up to the beginning of the path, given that the path starts in state z_m; 3) the probability of the observations between m and $m+n$ given the path; 4) the probability of the path itself; and 5) the probability of the observations from $m+n+1$ up to the end of the transcript, given that the path ends in state z_{m+n}. Replacing the terms in the above equation with variables gives us the expression:

$$\Pr(y, z|\theta) = \Big[\prod_{t=1}^{m-1} c_t\Big] \cdot \alpha_{z_m,m} \cdot \Big[\prod_{t=m+1}^{m+n} b_{z_t,t}\Big] \cdot \Big[\prod_{t=m+1}^{m+n} a_{z_{t-1},z_t}\Big] \cdot \beta_{z_{m+n},m+n} \quad (22)$$

We could normalize the joint probability with respect to all possible paths in the interval m to $m+n$, which is given by $\prod_{t=m}^{m+n} c_t$. However this would result in two biases. First, many paths are not feasible structures, effectively resulting in hyper-inflation of the joint probability with respect to path z. Second, path z will be penalized if alternative paths exist, which are significantly more likely given the data. As we are interested in identifying the putative location of a specific path, and specifically because we are *not* asking what the most likely path within the interval is (which is obtained using either the Viterbi algorithm or by maximum *a posteriori* estimation), we would like to normalize the joint probability of the observation and path z in a more appropriate manner. We can use the fact that our model is binary in terms of hidden states, implying that, with respect to a single path z, there exists only a single opposite path. We denote the opposite path by z'. In our hairpin example, $z' = \{0,0,0,0,1,1,1,0,0,0,0\}$. The joint probability of the data and path z', $\Pr(y, z'|\theta)$, can be expressed similarly to equation (22). If we take the log-ratio between these joint probabilities, we obtain a score that we denote:

$$\texttt{score}(z) = \log \frac{\Pr(y, z|\theta)}{\Pr(y, z'|\theta)},$$

which is expressed as

$$\frac{\Pr(y, z|\theta)}{\Pr(y, z'|\theta)} = \frac{\Big[\prod_{t=1}^{m-1} c_t\Big] \cdot \alpha_{z_m,m} \cdot \Big[\prod_{t=m+1}^{m+n} b_{z_t,t}\Big] \cdot \Big[\prod_{t=m+1}^{m+n} a_{z_{t-1},z_t}\Big] \cdot \beta_{z_{m+n},m+n}}{\Big[\prod_{t=1}^{m-1} c_t\Big] \cdot \alpha_{z'_m,m} \cdot \Big[\prod_{t=m+1}^{m+n} b_{z'_t,t}\Big] \cdot \Big[\prod_{t=m+1}^{m+n} a_{z'_{t-1},z'_t}\Big] \cdot \beta_{z'_{m+n},m+n}}$$
$$= \frac{\alpha_{z_m,m}}{\alpha_{z'_m,m}} \cdot \frac{\beta_{z_{m+n},m+n}}{\beta_{z'_{m+n},m+n}} \cdot \prod_{t=m+1}^{m+n} \frac{a_{z_{t-1},z_t} b_{z_t,t}}{a_{z'_{t-1},z'_t} b_{z'_t,t}}. \quad (23)$$

It should be noted that this definition of the score has the property of being equal to 0 when paths z and z' are equally likely within the considered region,

i.e. $\log \frac{\Pr(y,z|\theta)}{\Pr(y,z'|\theta)} = \log 1 = 0$. A positive score indicates that z is more likely than its inverse path z', while a negative score indicates the opposite. Note that we score motifs at each possible starting nucleotide and for each transcripts in the dataset unless sequence constraints are applied (see below).

Sequence constraints

Without sequence constraints, we score a motif starting at any possible nucleotide along a transcript, and this for all transcripts in the dataset. However, if the motif is self-contained, i.e. all base-pairings occur within a contiguous region, we can restrict the search space to only regions where sequences would allow for the motif to fold. We enforce such constraint by simply rejecting any regions where the transcript sequence does not permit the formation of Watson-Crick or Wobble base pairs between partners.

Viterbi path

The sequence of states that maximizes the likelihood of the data, $\mathcal{L}$, is found using the Viterbi algorithm and is thereby referred as the Viterbi path, V [2]. Briefly, the Viterbi path is determined by first filling a trellis matrix δ and a back pointer matrix ψ, followed by a backtracking of the optimal path as described below:

`Initialization:`

$$\delta_{i,1} = \log(\pi_i) + \log(b_{i,1})$$

$$\omega_{i,1} = 0$$

`Recursion:`

$$\delta_{j,t} = \max_{1 \le i \le N} \left[\delta_{i,t-1} + \log(a_{i,j}) \right] + \log(b_{j,t}) \ , \qquad 2 \le t \le T$$

$$\psi_{j,t} = \arg\max_{1 \le i \le N} \left[\delta_{i,t-1} + \log(a_{i,j}) \right] \ , \qquad 2 \le t \le T$$

`Termination and backtracking:`

$$V_T = \arg\max_{1 \le i \le N} [\omega_{i,T}]$$

$$V_t = \psi_{t+1}(V_{t+1}) \ , \qquad t = [T-1, T-2, \ldots, 1]$$

$$\texttt{with } 1 \le i \le N \tag{24}$$

Note that recursion values are log-transformed to avoid numerical overflow.

Pairing-state posterior probabilities

The posterior probability of state i at nucleotide t, denoted $\gamma_{i,t}$ is obtained by first computing emission probabilities as described in (8) using trained θ parameters. Using these emission we can then perform the forward-backward algorithm as described in (9). Finally, we can use equation (12) and sum over Gaussian components as described in (14) to obtain $\gamma_{i,t}$.

References

[1] Baum, L.E., Petrie, T., Soules, G., Weiss, N.: A maximization technique occurring in the statistical analysis of probabilistic functions of markov chains. The Annals of Mathematical Statistics **41**(1), 164–171 (1970). doi:10.1214/aoms/1177697196

[2] Rabiner, L.R.: A tutorial on hidden markov models and selected applications in speech recognition. Proceedings of the IEEE **77**(2), 257–286 (1989). doi:10.1109/5.18626

[3] Sükösd, Z., Swenson, M.S., Kjems, J., Heitsch, C.E.: Evaluating the accuracy of SHAPE-directed RNA secondary structure predictions. Nucleic Acids Research **41**(5), 2807–2816 (2013). doi:10.1093/nar/gks1283

[4] Deng, F., Ledda, M., Vaziri, S., Aviran, S.: Data-directed RNA secondary structure prediction using probabilistic modeling. RNA **22**(8), 1109–1119 (2016). doi:10.1261/rna.055756.115

[5] Yu, S.-Z., Kobayashi, H.: A hidden semi-markov model with missing data and multiple observation sequences for mobility tracking. Signal Processing **83**(2), 235–250 (2003). doi:10.1016/s0165-1684(02)00378-x

[6] Rahimi, A.: An Erratum for "A Tutorial on Hidden Markov Models and Selected Applications in Speech Recognition". http://alumni.media.mit.edu/~rahimi/rabiner/rabiner-errata/rabiner-errata.html (2010)
