# Supplementary material for: PATTERNA: transcriptome-wide search for functional RNA elements via structural data signatures
Source: Genome Biol. 2018 Mar 1;19:28. doi: 10.1186/s13059-018-1399-z (PMC5833111; doi:10.1186/s13059-018-1399-z)
Supplement: Supplementary file 2 — Supplementary Figures and Tables. (PDF 7600 kb) [file 13059_2018_1399_MOESM2_ESM.pdf]

Supplementary Figures and Tables for "patteRNA: transcriptome-wide search for functional RNA elements via structural data signatures"

Mirko Ledda and Sharon Aviran

Department of Biomedical Engineering and Genome Center, University of California at Davis, USA.

Runtime benchmarks

To assess computational requirements for mining motifs in transcriptome-wide datasets, we simulated datasets of varying sizes featuring diverse transcript lengths. Transcripts' sequences were simulated using a uniform nucleotide model (p=0.25 for A/C/G/U). SHAPE profiles were simulated in a two-stage process. First, we generated a sequence of pairing states using an HMM with parameters learned by *patteRNA* from the Weeks set. We then randomly sampled SHAPE values as described in [1] by using density functions proposed in [2] and fitting them to the Weeks set. Datasets consisting of various numbers of transcripts were simulated in a similar manner, with transcripts' lengths sampled without replacement from lengths observed in the PARS dataset [3]. Note that we restricted transcripts' lengths to be $\leq$ 5000 nt as we observed large variances in runtimes between repeated datasets with NNTM methods when very large transcripts were present.
We compared *patteRNA* to MFE and ensemble-sampling methods. For MFE, we used RNAfold (ViennaRNA package [4, 5]) and Fold-smp (RNAstructure package [6]). For ensemble-sampling, we sampled 1000 structures using GTfold [7], RNAsubopt (ViennaRNA package) and partition-smp/stochastic-smp (RNAstructure package). Note that as data-directed ensemble sampling is not currently implemented in the ViennaRNA package, we used predictions based on sequence alone with RNAsubopt. Executed commands are reported in Table S4. All benchmarks were run on a server (Ubuntu 14.04.1) and each session was allocated 16 CPUs and 16GB of RAM. We performed 5 repeats for each method and each dataset when varying RNA lengths (Figure S3A-B). Average wall times are reported and data were fitted using the following exponential growth function with x denoting a single transcript length:

$$\texttt{estimated runtime} = ax^b + c,\ a > 0,\ b > 0,\ c > 0 \quad (1)$$

Fitted parameters are available in Table S5. For simulated datasets containing varying number of transcripts, we performed 2 repeats for each method and each dataset and plotted predicted runtimes (Figure S3C-D). These predictions (marked by crosses in panel C) were obtained by computing the runtime required for each transcript individually using equation (1) and the parameters listed in Table S5. We then obtained a predicted runtime for an entire dataset by summing over all transcripts. We further applied the same approach to estimate runtime for the PARS dataset and two human GRCh38 (hg19) examples (see Table S7). The composition of these transcriptome-wide datasets are available in Table S6.

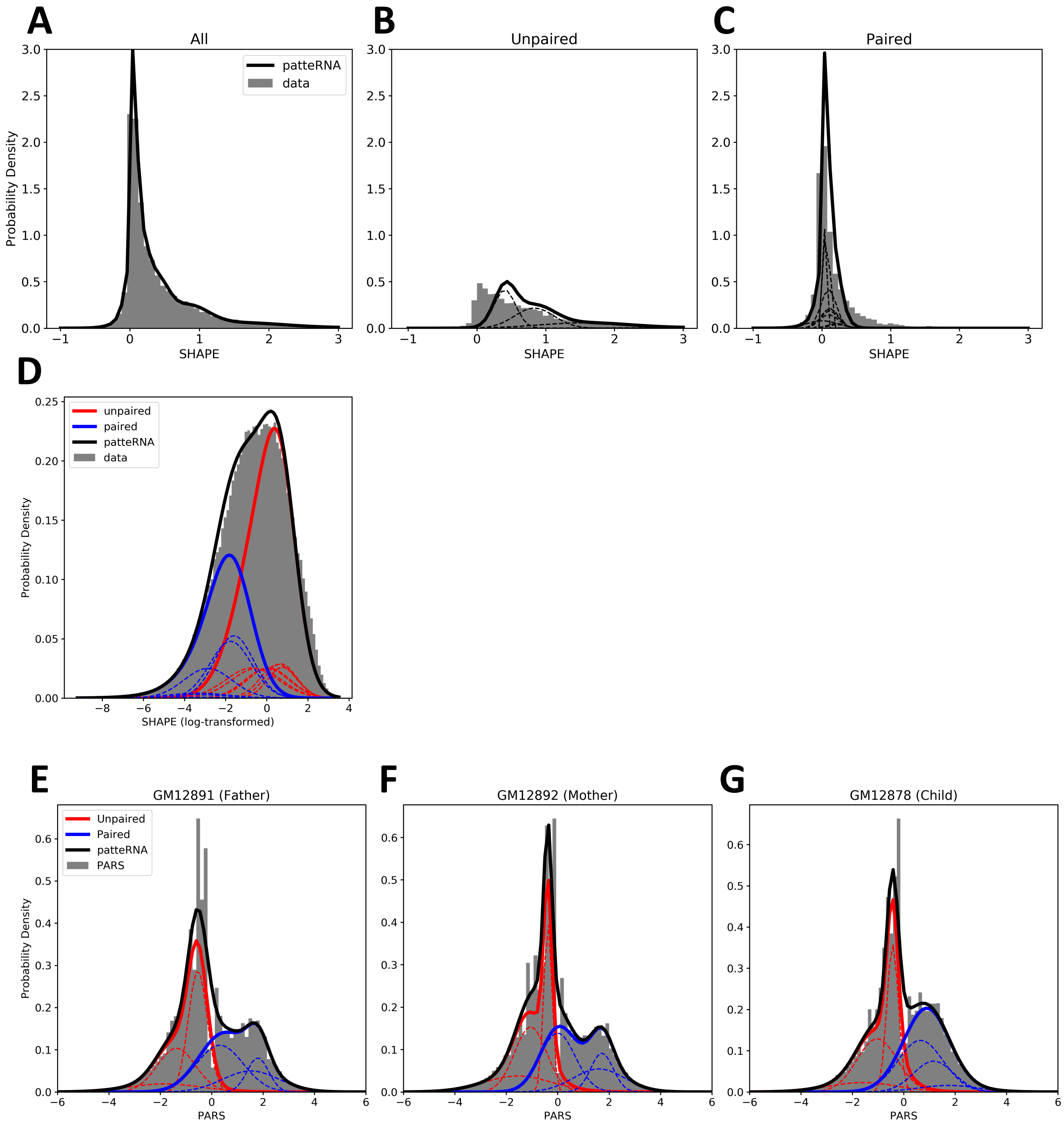

Figure S1: *patteRNA* trained on multiple datasets. Gaussian Mixture Models (black line) learned by *patteRNA* using SHAPE data from the Weeks set **(A-C)**, log-transformed SHAPE data from the fluoride riboswitch set **(D)** and PARS data from father **(E)**, mother **(F)** and child datasets **(G)**. Grey histograms represent the distribution of the data. For the Weeks set **(A)**, reactivities were subsequently broken down into each pairing state using reference structures to assess *patteRNA*'s state-dependent models accuracy at unpaired **(B)** and paired **(C)** nucleotides. **(D-G)** Pairing-state-dependent distributions are shown (solid colored lines). Individual Gaussian components are highlighted by dashed black/colored lines.

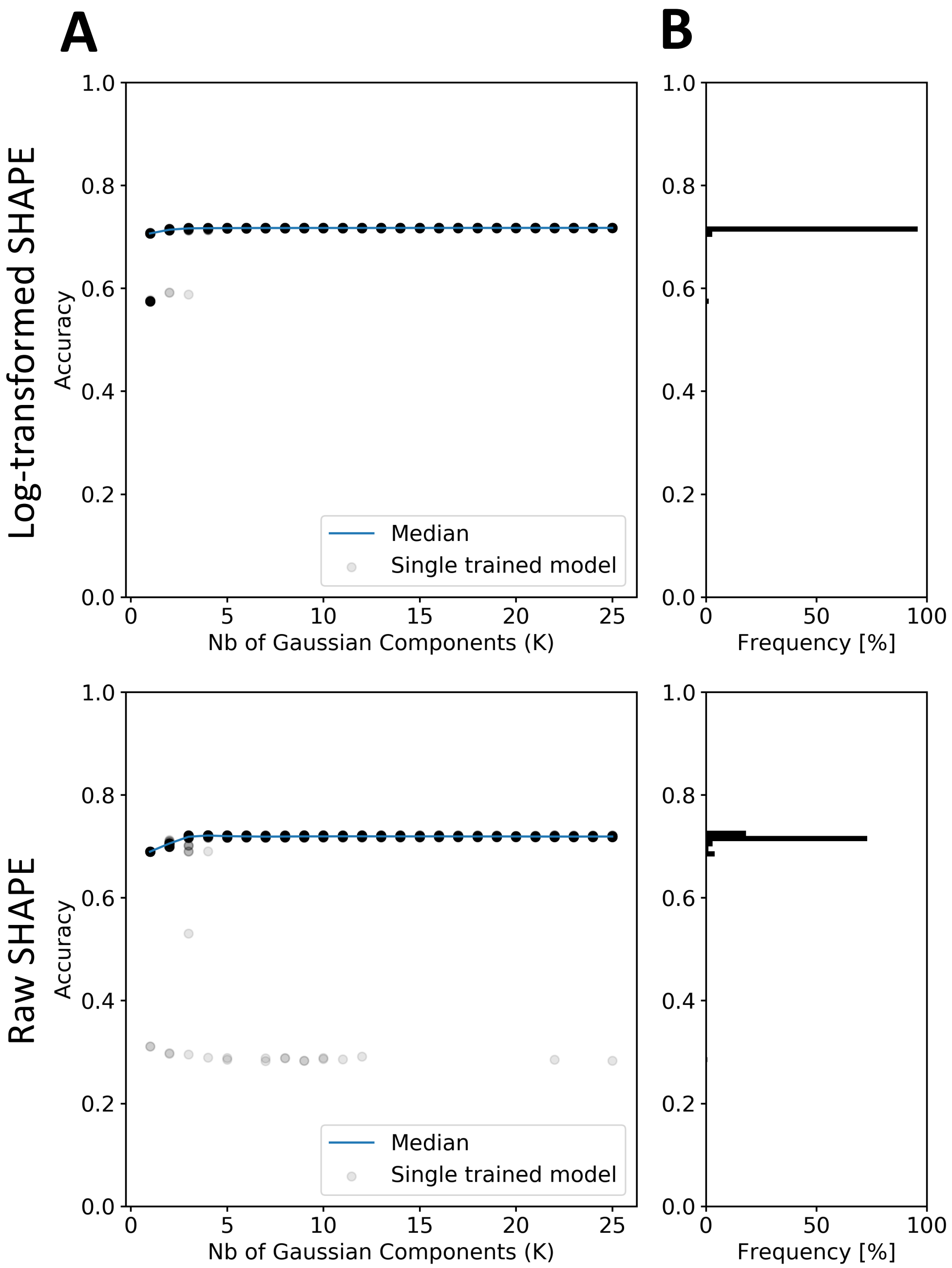

Figure S2: ***patteRNA*** **is robust to parameters choice and initialization on SHAPE data.** To assess the robustness of *patteRNA*, we varied the number of Gaussian kernels used per pairing states from 1 to 25 components (K). For each K, we randomly initialized the components and performed 100 training repetitions. We used each trained model to compute pairing state probabilities and subsequently, prediction accuracy relative to known reference structures. We tested both log-transformed and raw SHAPE input data. **(A)** Each semi-transparent (alpha=0.1) data point represent the accuracy obtained in an individual training repeat. Blue line corresponds to the median across repeats for each K. **(B)** Horizontal histogram highlights the frequency of accuracy values obtained across Gaussian components and repeats.

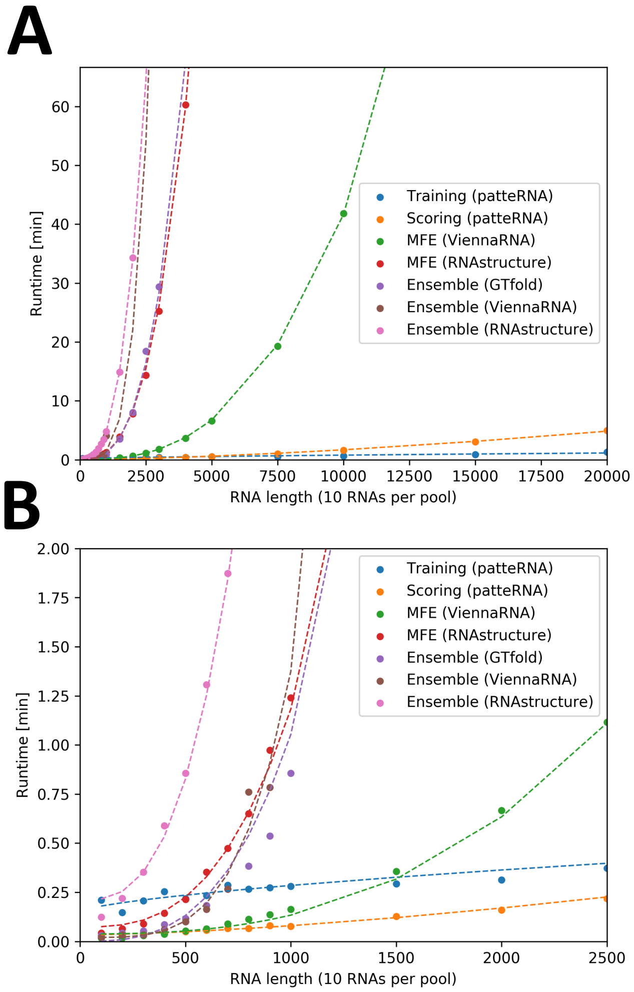

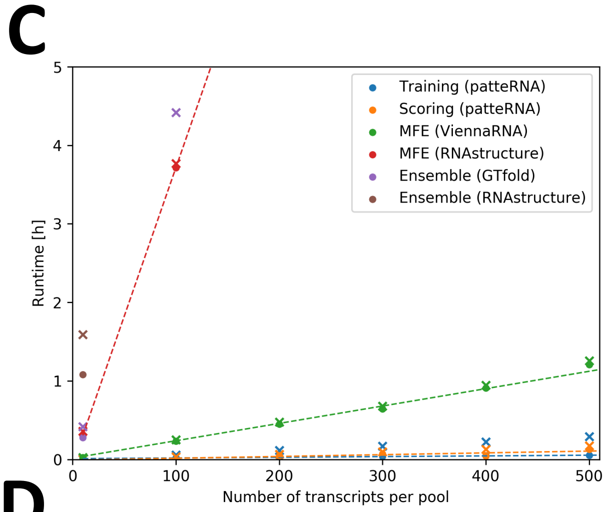

D

# Transcripts	Rep	Transcripts' lengths Median	Min	Max
10	1	2362.5	229	5000
10	2	986	99	3012
100	1	1321	63	5000
100	2	1827	67	5000
200	1	1470	28	5000
200	2	1396	56	5000
300	1	1332	64	5000
300	2	1345	61	5000
400	1	1237	50	5000
400	2	1203.5	30	5000
500	1	1491	57	5000
500	2	1682	58	5000

Figure S3: *patteRNA* run time is linear with respect to RNA length. (A-B) Runtimes to process small datasets of 10 RNAs of increasing lengths. Datapoints represent average wall times over 5 repeats. An exponential model (see section "Runtime benchmarks") was fitted to the data and is represented by the dashed line. Note that only processes finishing within 2h were considered. **(B)** Zoomed-in view of panel A for short RNAs (≤ 2500) **(C)** Runtimes for simulated datasets composed of varying numbers of transcripts of different lengths, with length capped at 5000 nt. Datapoints represent average wall times over 2 repeats. Crosses represent estimated runtimes using an exponential model (see section "Runtime benchmarks") with parameters listed in Table S5. A linear model was fitted to the data and is represented by dashed lines. Note that only processes finishing within 5h were considered. For instance, while RNAsubopt was tested it is not shown as the process took over 5h for the smallest set of 10 RNAs. **(D)** Summary of dataset compositions in terms of transcripts' lengths plotted in panel C. Commands used with each method are reported in Table S4.

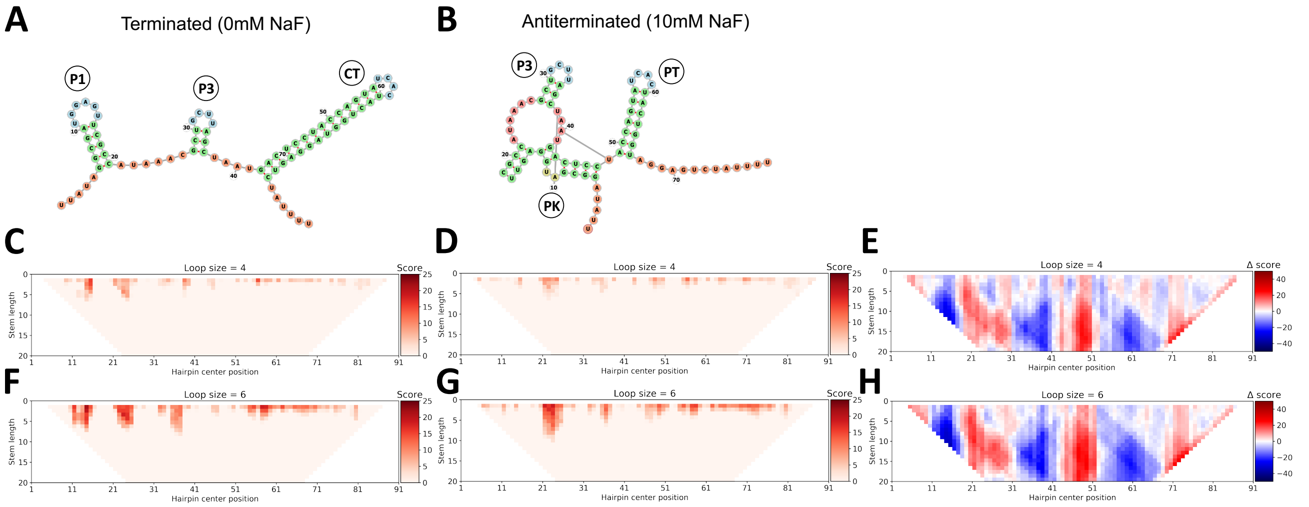

Figure S4: Motif detection in a bi-stable fluoride riboswitch. **(A)** The accepted structure of the terminated fold, associated with "transcription off" state, features three hairpins: P1, P3 and CT (complete terminator). **(B)** The accepted structure of the anti-terminated fold, associated with "transcription on" state, features a pseudoknot domain (PK) and a partial terminator (PT) hairpin. Pseudo-hairpins of loop-size 4 **(C-D)** and 6 **(F-G)**, and variable stem-size were scored across all possible starting nucleotides of the full-length transcript (100 nt) and with no sequence constraints (hence the term pseudo-hairpin). X-axis indicates the position of the motif's center. Y-axis corresponds to hairpins with stems of variable lengths. Reds indicate higher scores. **(E-H)** Differential scores between fluoride conditions for pseudo-hairpins of loop-size **(E)** 4 and **(H)** 6. A red color indicates that the data better supports the presence of pseudo-hairpins in the presence of fluoride, and inversely, blue in the absence of fluoride.

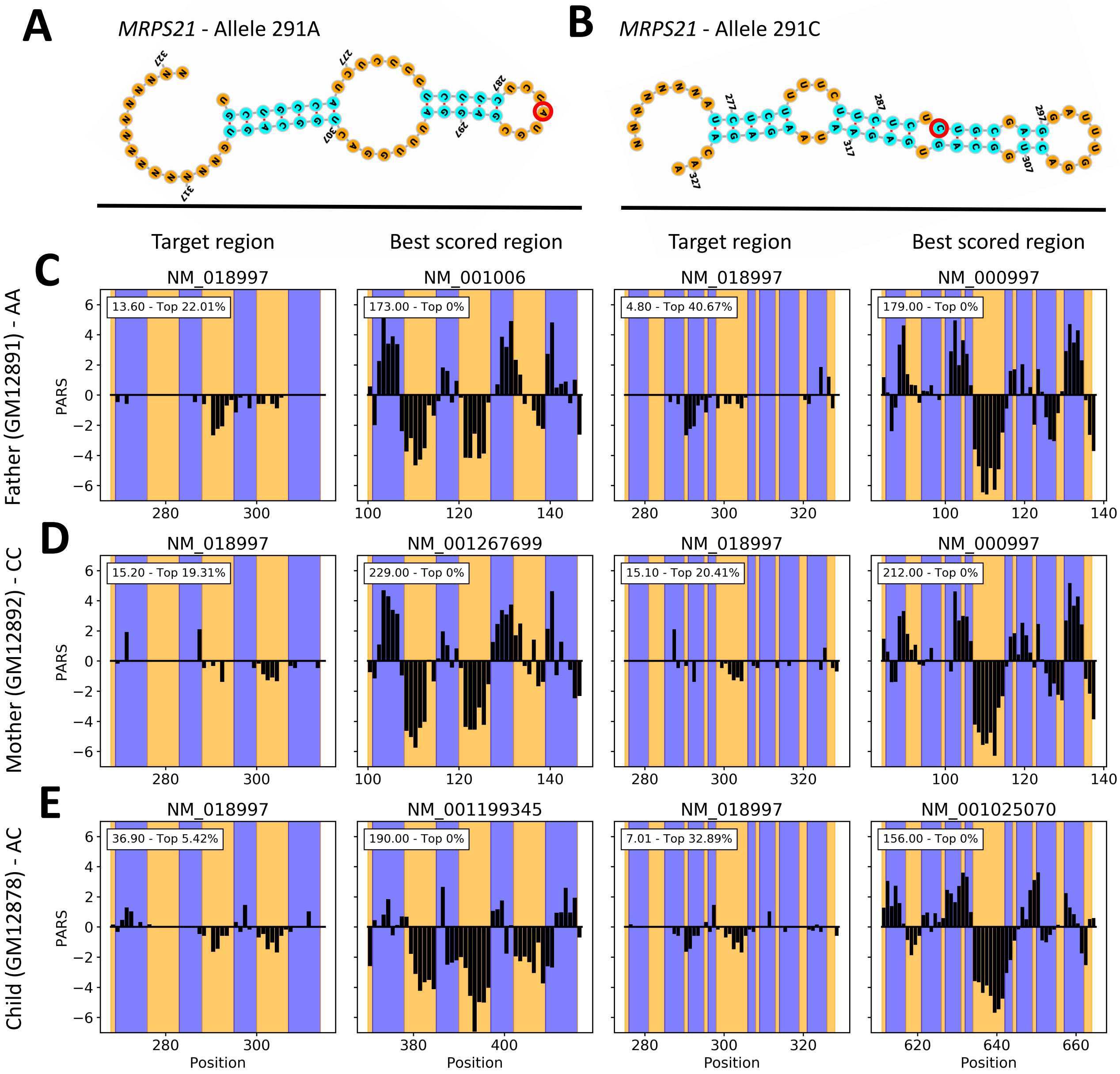

Figure S5: Transcriptome-wide search for the *MRPS21* riboSNitch motif in PARS data. Secondary structure models proposed in Wan *et al.* [3] for allele variants 291A **(A)** and 291C **(B)** of the *MRPS21* riboSNitch. Red circles highlight the single nucleotide polymorphism and N's indicate nucleotides that were added to maintain the same sequence range between the two allelic variants but were not used when scoring motifs as they are described in Wan *et al.*. Search results obtained for the father (homozygote A) **(C)**, mother (homozygote C) **(D)** and child (heterozygote) **(E)** datasets. For each riboSNitch variant, PARS traces at both the target location, i.e., the location where the riboSNitch was first reported, and the best scoring location across tested transcripts are shown. Blue regions indicate helices, i.e. paired nucleotides where positive PARS values are expected; and inversely for orange regions. The inset reports both the score and rank of the scored region relative to all scored regions, where a smaller rank indicates a region is among the best scored ones, with 0% indicating the top scored region.

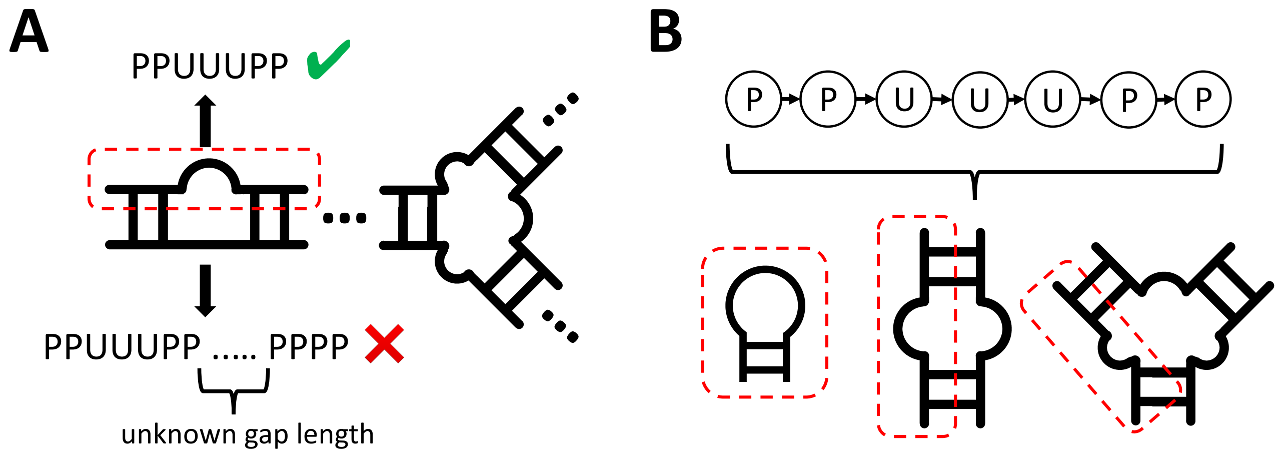

Figure S6: Overview of valid motifs and limitations of *patteRNA*. **(A)** Because of combinatorial limitations, *patteRNA* cannot score non self-contained motifs. For instance, the region highlighted by the red dashed box is a valid target motif as its entire path (P=paired / U=unpaired nucleotides) does not contain any gap of unknown length. Inversely, the double-stranded bulge motif is invalid as it entails a gap in its pairing state path. **(B)** Illustration of a single state-path that could be produced from three distinct structural motifs. The red dashed boxes highlight example regions that would generate this path. Note that loops are 3 nt long and not drawn to scale.

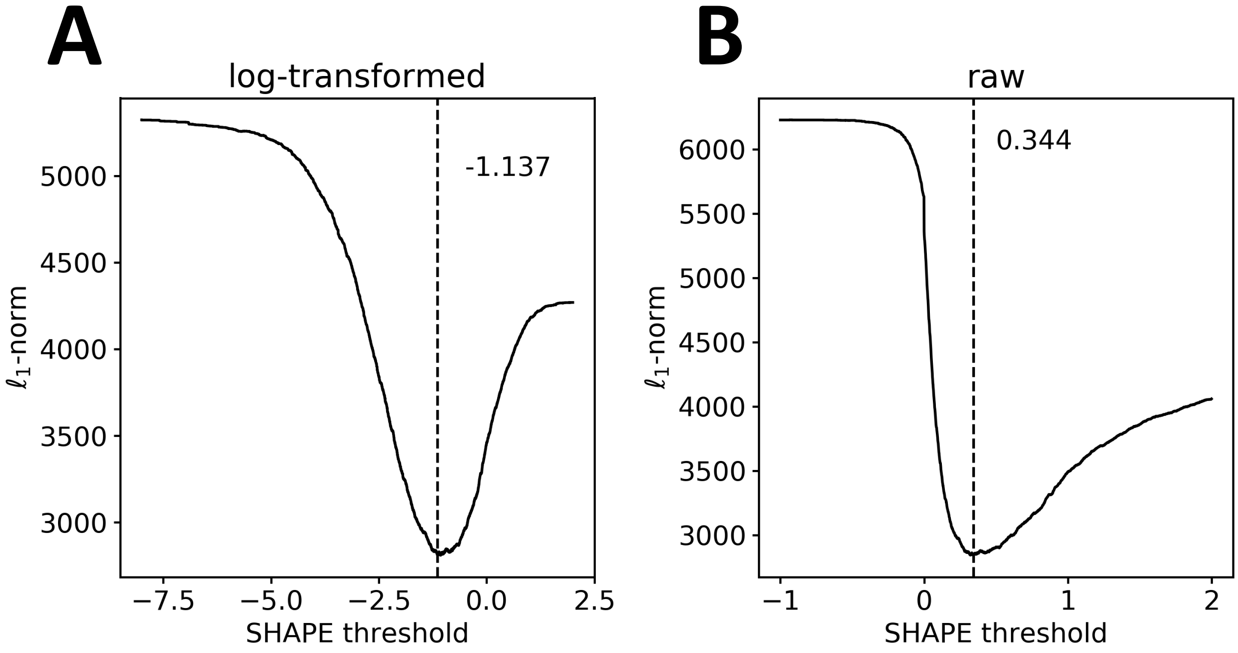

Figure S7: Optimal cutoff for a SHAPE-based classifier informed by reference structures. For each tested cutoff value, reactivities greater than the threshold were deemed unpaired and inversely for paired nucleotides. Predictions were then compared to reference structures to compute the ℓ_1-norm. Threshold optimization was performed on log-transformed (from -8 to 2 by 0.001) **(A)** and raw (from -1 to 2 by 0.001) SHAPE data from the Weeks set **(B)**. The optimal cutoff (vertical dashed line) was selected at the SHAPE value that minimizes the ℓ_1-norm.

Table S1: List of SHAPE profiles included in the Weeks set.

RNA	Length	Source
Pre-Q1 riboswitch, *B. subtilis*	34	[8]
Fluoride riboswitch, *P. syringae*	66	[8]
Adenine riboswitch, *V. vulnificus*	71	[8]
tRNA(asp), *yeast*	75	[9]
tRNA(phe), *E. coli*	76	[8]
TPP riboswitch, *E. coli*	79	[8]
cyclic-di-GMP riboswitch, *V. cholerae*	97	[8]
SAM I riboswitch, *T. tengcongensis*	118	[8]
5S rRNA, *E. coli*	120	[8]
M-Box riboswitch, *B. subtilis*	154	[8]
P546 domain, bI3 group I intron	155	[9]
Lysine riboswitch, *T. maritima*	174	[8]
Group I intron, *Azoarcus sp.*	214	[8]
Hepatitis C virus IRES domain	336	[8]
Group II intron, *O. iheyensis*	412	[8]
Group I Intron, *T. thermophila*	425	[8]
5′ domain of 23S rRNA, *E. coli*	511	[8]
16S rRNA, *H. volcanii*	1474	[10]
16S rRNA, *C. difficile*	1503	[10]
16S rRNA, *E. coli*	1542	[9]
23S rRNA, *E. coli*	2904	[9]

Table S2: Search results in the child dataset spiked with the *MRPS21* motif harboring "perfect" PARS information.

Spiked	Searched	Rank	Total	p
A	A	0	1805472	0
A	C	378717	1798458	0.211
C	A	377228	1805472	0.209
C	C	0	1798458	0

Table S3: Accessions for the fluoride riboswitch and the PARS sets used in this study.

Sample	Probing	Experiment	Accession code	Database
F^- riboswitch	SHAPE-seq	0mM NaF (rep. 1)	FLUORSW_BZCN_0001	RMDB database
F^- riboswitch	SHAPE-seq	0mM NaF (rep. 2)	FLUORSW_BZCN_0002	
F^- riboswitch	SHAPE-seq	0mM NaF (rep. 3)	FLUORSW_BZCN_0003	
F^- riboswitch	SHAPE-seq	10mM NaF (rep. 1)	FLUORSW_BZCN_0004	
F^- riboswitch	SHAPE-seq	10mM NaF (rep. 2)	FLUORSW_BZCN_0005	
F^- riboswitch	SHAPE-seq	10mM NaF (rep. 3)	FLUORSW_BZCN_0006	
GM12891 (Father)	PARS	V1 counts	GSM1226159	GEO database, accession GSE50676
	PARS	S1 counts	GSM1226160	
GM12892 (Mother)	PARS	V1 counts	GSM1226161	
	PARS	S1 counts	GSM1226162	
GM12878 (Child)	PARS	V1 counts	GSM1226157	
	PARS	S1 counts	GSM1226158	

Table S4: Commands used for the runtime benchmark ($FOOBAR indicate variables).

Method	Command
training (patteRNA)	patteRNA $SHAPE $OUTPUT -f $FASTA -lv -k 10
scoring (patteRNA)	patteRNA $SHAPE $OUTPUT -f $FASTA -lv –model $MODEL –gammas –viterbi –pattern "((((....))))"
ensemble (GTfold)	gtboltzmann –useSHAPE $SHAPE $FASTA -o $OUTPUT -p $PARAM_DIR -s 1000 –estimatebpp –verbose –bpp
MFE (ViennaRNA)	RNAfold –shape=$SHAPE < $FASTA > $OUTPUT
ensemble (ViennaRNA)	RNAsubopt < $FASTA > $OUTPUT
MFE (RNAstructure)	Fold-smp $FASTA -sh $SHAPE $OUT
ensemble (RNAstructure)	partition-smp $FASTA -sh $SHAPE $PFS + stochastic-smp $PFS $OUTPUT

Table S5: Parameters of the exponential model used to fit method's runtimes

Method	Parameters		
	**a**	**b**	**c**
training (patteRNA)	7.98e-03	0.67	0.91
scoring (patteRNA)	6.46e-06	1.55	0.20
MFE (ViennaRNA)	7.02e-09	2.64	0.23
MFE (RNAstructure)	1.59e-08	2.87	0.44
Ensemble (GTfold)	6.32e-09	3.00	1.00e-10
Ensemble (ViennaRNA)	7.18e-12	4.02	0.12
Ensemble (RNAstructure)	5.96e-08	2.89	1.26

Table S6: Number of transcripts and lengths in example transcriptome-wide SP dataset

Dataset	# Transcripts	Median length	Min	Max	Source
PARS	74210	1427	8	109223	[3]
PARS (high cov)*	2511	1537	54	12495	[3]
GRCh38 cDNAs	180868	886	8	109224	Ensembl[a]
GRCh38 ncDNAs	37296	554	5	205012	Ensembl[b]

* High coverage refers to the 2012 transcripts from the child dataset that passed quality control filtering (see section "Motif searches in transcriptome-wide PARS data" in Methods).

[a] ftp://ftp.ensembl.org/pub/release-90/fasta/homo_sapiens/cdna/Homo_sapiens.GRCh38.cdna.all.fa.gz

[b] ftp://ftp.ensembl.org/pub/release-90/fasta/homo_sapiens/ncrna/Homo_sapiens.GRCh38.ncrna.fa.gz

Table S7: Approximate runtimes for various transcriptome-wide datasets.

Method	Dataset			
	**PARS**	**PARS (high cov)**	**GRCh38 cDNAs**	**GRCh38 ncRNAs**
training (patteRNA)	43 h	1.5 h	4 days	16 h
scoring (patteRNA)	30 h	46 min	53 h	6 h
MFE (ViennaRNA)*	3 weeks	5.5 h	1 month	12 days
MFE (RNAstructure)*	>1 year	90 h	>1 year	>1 year
Ensemble (GTfold)*	>1 year	4 days	>1 year	>1 year
Ensemble (ViennaRNA)*	>1 year	1 month	>1 year	>1 year
Ensemble (RNAstructure)*	>1 year	2 weeks	>1 year	>1 year

* Note that these runtimes are only to generate structure predictions and do not include the search for a motif.

References

[1] Deng, F., Ledda, M., Vaziri, S., Aviran, S.: Data-directed RNA secondary structure prediction using probabilistic modeling. RNA **22**(8), 1109–1119 (2016). doi:10.1261/rna.055756.115

[2] Sükösd, Z., Swenson, M.S., Kjems, J., Heitsch, C.E.: Evaluating the accuracy of SHAPE-directed RNA secondary structure predictions. Nucleic Acids Research **41**(5), 2807–2816 (2013). doi:10.1093/nar/gks1283

[3] Wan, Y., Qu, K., Zhang, Q.C., Flynn, R.A., Manor, O., Ouyang, Z., Zhang, J., Spitale, R.C., Snyder, M.P., Segal, E., Chang, H.Y.: Landscape and variation of RNA secondary structure across the human transcriptome. Nature **505**(7485), 706–709 (2014). doi:10.1038/nature12946

[4] Lorenz, R., Bernhart, S.H., Hner Zu Siederdissen, C., Tafer, H., Flamm, C., Stadler, P.F., Hofacker, I.L.: Viennarna package 2.0. Algorithms for molecular biology : AMB **6**, 26 (2011). doi:10.1186/1748-7188-6-26

[5] Lorenz, R., Luntzer, D., Hofacker, I.L., Stadler, P.F., Wolfinger, M.T.: SHAPE directed RNA folding. Bioinformatics **32**(1), 145–147 (2016). doi:10.1093/bioinformatics/btv523

[6] Reuter, J.S., Mathews, D.H.: RNAstructure: software for RNA secondary structure prediction and analysis. BMC Bioinformatics **11**(1), 129 (2010). doi:10.1186/1471-2105-11-129

[7] Swenson, M.S., Anderson, J., Ash, A., Gaurav, P., Sükösd, Z., Bader, D.A., Harvey, S.C., Heitsch, C.E.: GTfold: Enabling parallel RNA secondary structure prediction on multi-core desktops. BMC Research Notes **5**(1), 341 (2012). doi:10.1186/1756-0500-5-341

[8] Hajdin, C.E., Bellaousov, S., Huggins, W., Leonard, C.W., Mathews, D.H., Weeks, K.M.: Accurate SHAPE-directed RNA secondary structure modeling, including pseudoknots. Proceedings of the National Academy of Sciences **110**(14), 5498–5503 (2013). doi:10.1073/pnas.1219988110

[9] Deigan, K.E., Li, T.W., Mathews, D.H., Weeks, K.M.: Accurate SHAPE-directed RNA structure determination. Proceedings of the National Academy of Sciences **106**(1), 97–102 (2008). doi:10.1073/pnas.0806929106

[10] Lavender, C.A., Lorenz, R., Zhang, G., Tamayo, R., Hofacker, I.L., Weeks, K.M.: Model-free RNA sequence and structure alignment informed by SHAPE probing reveals a conserved alternate secondary structure for 16s rRNA. PLOS Computational Biology **11**(5), 1004126 (2015). doi:10.1371/journal.pcbi.1004126
