# Supplementary material for: PATTERNA: transcriptome-wide search for functional RNA elements via structural data signatures
Source: Genome Biol. 2018 Mar 1;19:28. doi: 10.1186/s13059-018-1399-z (PMC5833111; doi:10.1186/s13059-018-1399-z)
Supplement: Supplementary file 3 — Reviewer reports and author’s response to reviewers. (DOCX 21 kb) [file 13059_2018_1399_MOESM3_ESM.docx]

**Open Peer Review reports**

Pre-publication versions of this article and author comments to reviewers are available by contacting [info@biomedcentral.com](mailto:info@biomedcentral.com).

| Original submission |  |  |
| --- | --- | --- |
| 05 September 2017 | Submitted | Original manuscript |
| 19 September 2017 | Reviewed | Reviewer report - [Daniel Blockmans](javascript:popupReviewerInfo(446,81)) |
| 27 September 2017 | Reviewed | Reviewer report - [Sarah Mackie](javascript:popupReviewerInfo(324,81)) |
| 03 October 2017 | Author responded | Author comments - Matthew Koster |
| Resubmission – Version 2 |  |  |
| 03 October 2017 | Submitted | Manuscript version 2 |
| Publishing |  |  |
| 19 October 2017 | Editorially accepted |  |
| 28 November 2017 | Article published | 10.1186/s41927-017-0004-5 |

### How does Open Peer Review work?

Open peer review is a system where authors know who the reviewers are, and the reviewers know who the authors are. If the manuscript is accepted, the named reviewer reports are published alongside the article. Pre-publication versions of the article and author comments to reviewers are available by contacting [info@biomedcentral.com](mailto:%20mailto:info@biomedcentral.com). All previous versions of the manuscript and all author responses to the reviewers are also available.

You can find further information about the peer review system at <https://bmcrheumatol.biomedcentral.com/about#peer-review+policy>

**ORIGINAL SUBMISSION**

**Reviewer’s report: Reviewer 1**

**Title:** Giant cell arteritis: pathogenic mechanisms and new potential therapeutic targets

**Version**: 0	**Date**: 19 Sept 2017

**Reviewer**:	Daniel Blockmans

**Reviewer's report:**

Excellently written review, just perfect. 
I miss however a paragraph on the function of vascular smooth muscle cells and the endothial ceels in intimal hyperplasia in GCA (see for instance the recent report of the group of Maria Cid on the role of endothelin-1 in vascular smooth muscle migration (Ann Rheum Dis 2017; 76: 1623-1633))

**Quality of written English**

Please indicate the quality of language in the manuscript:

Acceptable

**Declaration of competing interests**

Please complete a declaration of competing interests, considering the following questions:

1. Have you in the past five years received reimbursements, fees, funding, or salary from an organisation that may in any way gain or lose financially from the publication of this manuscript, either now or in the future?

2. Do you hold any stocks or shares in an organisation that may in any way gain or lose financially from the publication of this manuscript, either now or in the future?

3. Do you hold or are you currently applying for any patents relating to the content of the manuscript?

4. Have you received reimbursements, fees, funding, or salary from an organization that holds or has applied for patents relating to the content of the manuscript?

5. Do you have any other financial competing interests?

6. Do you have any non-financial competing interests in relation to this paper?

If you can answer no to all of the above, write 'I declare that I have no competing interests' below. If your reply is yes to any, please give details below.

I declare that I have no competing interests

I agree to the open peer review policy of the journal. I understand that my name will be included on my report to the authors and, if the manuscript is accepted for publication, my named report including any attachments I upload will be posted on the website along with the authors' responses.

I agree for my report to be made available under an Open Access Creative Commons CC-BY license (http://creativecommons.org/licenses/by/4.0/). I understand that any comments which I do not wish to be included in my named report can be included as confidential comments to the editors, which will not be published.

I agree to the open peer review policy of the journal

**Reviewer’s report: Reviewer 2**

Title: Giant cell arteritis: pathogenic mechanisms and new potential therapeutic targets

**Version**: 0	**Date**: 27 Sept 2017

**Reviewer**:	Sarah Mackie

**Reviewer's report:**

This is a thorough and excellent "state of the art" review of giant cell arteritis, with a focus on therapeutic targets.

For completeness, the authors might consider mentioning the recently-published nested case-control study (Rhee et al., Ann Rheum Dis 2017) with regard to the question of infectious trigger including varicella zoster virus.

**Quality of written English**

Please indicate the quality of language in the manuscript:

Acceptable

**Declaration of competing interests**

Please complete a declaration of competing interests, considering the following questions:

1. Have you in the past five years received reimbursements, fees, funding, or salary from an organisation that may in any way gain or lose financially from the publication of this manuscript, either now or in the future?

2. Do you hold any stocks or shares in an organisation that may in any way gain or lose financially from the publication of this manuscript, either now or in the future?

3. Do you hold or are you currently applying for any patents relating to the content of the manuscript?

4. Have you received reimbursements, fees, funding, or salary from an organization that holds or has applied for patents relating to the content of the manuscript?

5. Do you have any other financial competing interests?

6. Do you have any non-financial competing interests in relation to this paper?

If you can answer no to all of the above, write 'I declare that I have no competing interests' below. If your reply is yes to any, please give details below.

Atttendance at Medical Advisory Board meetings - Roche/Chugai; Sanofi

I agree to the open peer review policy of the journal. I understand that my name will be included on my report to the authors and, if the manuscript is accepted for publication, my named report including any attachments I upload will be posted on the website along with the authors' responses.

I agree for my report to be made available under an Open Access Creative Commons CC-BY license (http://creativecommons.org/licenses/by/4.0/). I understand that any comments which I do not wish to be included in my named report can be included as confidential comments to the editors, which will not be published.

I agree to the open peer review policy of the journal

**Author’s response to reviews**

**Title:** Giant cell arteritis: pathogenic mechanisms and new potential therapeutic targets

**Authors:**

Matthew J. Koster (koster.matthew@mayo.edu)

Kenneth J. Warrington, M.D. (warrington.kenneth@mayo.edu)

**Version**: 1	**Date**: 03 Oct 2017

**Author’s response to reviews:**

October 3, 2017

James Mockridge, PhD

Editor

*BMC Rheumatology*

Re: Manuscript ID BRHM-D-17-00018, “Giant cell arteritis: pathogenic mechanisms and new potential therapeutic targets”

Dear Professor Mockridge:

Thank you for reviewing our manuscript, “Giant cell arteritis: pathogenic mechanisms and new potential therapeutic targets” and for providing the opportunity to submit it with revisions for reconsideration for publication in *BMC Rheumatology*.

We thank the reviewers for their constructive comments and we have revised the manuscript accordingly. A point-by-point response to each comment is provided on the following page.

We hope that with these revisions, our manuscript will now be acceptable for publication in *BMC Rheumatology,* and we look forward to hearing from you.

Kind regards,

Matthew J. Koster, MD

Division of Rheumatology

Mayo Clinic

200 First Street SW

Rochester, MN 55905, USA

Email: Koster.Matthew@mayo.edu

Reviewer #1: Excellently written review, just perfect.

I miss however a paragraph on the function of vascular smooth muscle cells and the endothial ceels in intimal hyperplasia in GCA (see for instance the recent report of the group of Maria Cid on the role of endothelin-1 in vascular smooth muscle migration (Ann Rheum Dis 2017; 76: 1623-1633))

**Response:**

We agree with the reviewer that addition of information on endothelial cells would be of benefit and have added to the manuscript: (Section Main text, Subheading Endothelial cells, Lines 270-284)

Reviewer #2: This is a thorough and excellent "state of the art" review of giant cell arteritis, with a focus on therapeutic targets.

For completeness, the authors might consider mentioning the recently-published nested case-control study (Rhee et al., Ann Rheum Dis 2017) with regard to the question of infectious trigger including varicella zoster virus.

**Response:**
We agree with the reviewer that reference of the recent article by Rhee et al. would be of benefit and have added this to the manuscript (Section: Main Text, Subheading Infection, Lines 135-141)
